# Plasma-assisted oxidation of Cu(100) and Cu(111)†

**DOI:** 10.1039/d1sc04861a

**Published:** 2021-10-18

**Authors:** Sebastian Kunze, Liviu C. Tănase, Mauricio J. Prieto, Philipp Grosse, Fabian Scholten, Lucas de Souza Caldas, Dennis van Vörden, Thomas Schmidt, Beatriz Roldan Cuenya

**Affiliations:** Department of Interface Science, Fritz-Haber Institute of the Max Planck Society 14195 Berlin Germany roldan@fhi-berlin.mpg.de schmidtt@fhi-berlin.mpg.de; Department of Physics, Ruhr-University Bochum 44780 Bochum Germany

## Abstract

Oxidized copper surfaces have attracted significant attention in recent years due to their unique catalytic properties, including their enhanced hydrocarbon selectivity during the electrochemical reduction of CO_2_. Although oxygen plasma has been used to create highly active copper oxide electrodes for CO_2_RR, how such treatment alters the copper surface is still poorly understood. Here, we study the oxidation of Cu(100) and Cu(111) surfaces by sequential exposure to a low-pressure oxygen plasma at room temperature. We used scanning tunnelling microscopy (STM), low energy electron microscopy (LEEM), X-ray photoelectron spectroscopy (XPS), near edge X-ray absorption fine structure spectroscopy (NEXAFS) and low energy electron diffraction (LEED) for the comprehensive characterization of the resulting oxide films. O_2_-plasma exposure initially induces the growth of 3-dimensional oxide islands surrounded by an O-covered Cu surface. With ongoing plasma exposure, the islands coalesce and form a closed oxide film. Utilizing spectroscopy, we traced the evolution of metallic Cu, Cu_2_O and CuO species upon oxygen plasma exposure and found a dependence of the surface structure and chemical state on the substrate's orientation. On Cu(100) the oxide islands grow with a lower rate than on the (111) surface. Furthermore, while on Cu(100) only Cu_2_O is formed during the initial growth phase, both Cu_2_O and CuO species are simultaneously generated on Cu(111). Finally, prolonged oxygen plasma exposure results in a sandwiched film structure with CuO at the surface and Cu_2_O at the interface to the metallic support. A stable CuO(111) surface orientation is identified in both cases, aligned to the Cu(111) support, but with two coexisting rotational domains on Cu(100). These findings illustrate the possibility of tailoring the oxidation state, structure and morphology of metallic surfaces for a wide range of applications through oxygen plasma treatments.

## Abstract 2

A low-pressure oxygen plasma oxidized Cu(100) and Cu(111) surfaces at room temperature. The time-dependent evolution of surface structure and chemical composition is reported in detail for a range of exposure times up to 30 min.

## Graphical abstract

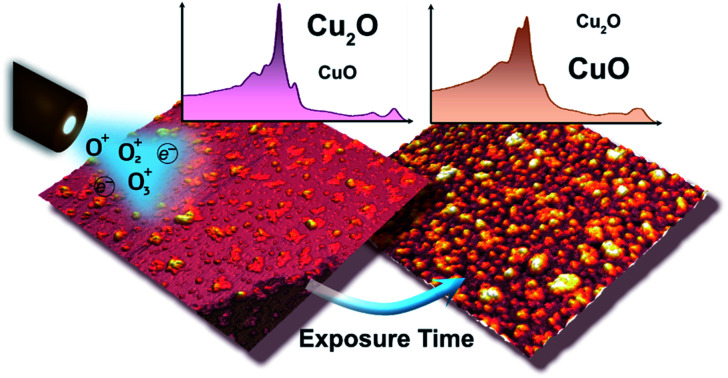

## Introduction

Copper has a long history of industrial applications in metallurgy, construction and electronics. Due to its widespread use, insights into the oxidation dynamics of copper are interesting for various science and technology fields. The main oxides of copper, Cu_2_O and CuO, are both p-type semiconductors and are themselves investigated for their application in solar cells^1,2^ and as photocatalysts.^3,4^ Furthermore, copper has unique properties that make it suitable for use as a catalyst in the electrochemical conversion of CO_2_ (CO_2_RR) to multicarbon products, such as ethylene.^5^ Several studies have shown that oxidizing the copper surfaces can enhance the catalytic activity and selectivity towards certain products. Electrochemical oxidation is often utilized for its morphology-altering capabilities to obtain rough surfaces in the form of oxide-derived copper (OD copper).^6–8^ Our group has also shown that treatment of copper foils with an oxygen plasma results in lower overpotential and enhanced selectivity towards ethylene.^9,10^ The origin of the enhanced catalytic properties of OD-copper is thought to lie in morphological transformations undergone by the pre-oxidized Cu surfaces during the reducing CO_2_RR conditions, as well as to the presence of resilient Cu(i) species that might remain at/near the surface during reaction.^11,12^ In fact, the coexistence of Cu(0)/Cu(i) species during CO_2_RR has been recently achieved through pulsed electrochemical treatments and shown to open up a new and highly selective route towards ethanol generation.^13^ The essential role of the morphology for the CORR and CO_2_RR has become apparent since studies on copper single crystals have shown selectivity deviations between Cu(111), which mainly produces methane, and Cu(100), which is selective for ethylene.^14,15^ Furthermore, our group has published a comprehensive study showing that surface structure is even more critical than previously assumed. This was demonstrated by the dependency of hydrocarbon formation on the prevalence of defects and roughness, when otherwise on ‘perfect’ atomically-clean flat surfaces only hydrogen was produced.^16^

Understanding how the oxide formation can be controlled and tuned is therefore a desirable goal for further research into efficient catalysts and energy materials. However, the complex nature of the processes involved has not yet been unravelled to a sufficient degree.^17^ In ultra-high vacuum, copper surfaces remain unreconstructed for an extended duration. The initial oxidation, on an atomic scale and under low oxygen pressures, depends on the orientation of the surface plane. On Cu(100), it has been shown that chemisorbed oxygen induces reconstructions of the surface even at low coverages.^18–20^ On the Cu(111) surface, ordered structures have not been reported at low oxygen coverages,^21,22^ while hexagonal overlayers have been identified for higher coverages.^23^ The formation of reconstructed O/Cu(111) surfaces by exposure to molecular oxygen requires higher temperatures, leading to several reconstructions, notably the “29” and “44” reconstructions.^24,25^ Owing to the requirement of flat surfaces, studies on the oxidized surface at the atomic scale utilized either very low oxygen exposures, high temperature oxidation (inducing reconstruction) or bulk Cu_2_O single crystals. This also lead to a lack of studies in the range between the initial oxide growth and later stages of oxide film growth, with only few studies tracking the propagation of the oxide growth over time.^26,27^

Notably absent from fundamental studies is the oxidation at room temperature (RT) *via* exposure to an oxygen plasma. Little research has been done on the morphology of such oxidized surfaces besides the identification of increased surface roughness.^28^ One of the most appealing aspects to use a plasma is the possibility of decoupling the oxidation process from elevated temperatures (thermal oxidation)^25^ and chemically compromising environments (electrooxidation). Here, we investigate the oxidation of Cu(111) and Cu(100) single-crystal surfaces with a low-pressure oxygen plasma at room temperature. We applied a comprehensive suite of complementary microscopic (STM, LEEM), spectroscopic (XPS, NEXAFS) and diffraction (LEED) techniques to study the growth dependencies in regards to the exposure and Cu surface orientation as a first step towards a deeper understanding of plasma-modified surfaces.

## Experimental

The experiments were performed in two separate UHV systems (called “STM/XPS system” and “LEEM/XPEEM” in the following) and with two sets of single crystals. Both UHV systems have a base pressure in the low 10^−10^ mbar range. The STM/XPS system is a commercial UHV system from SPECS GmbH used for STM (NAP-SPM 150 Aarhus) and XPS (monochromated X-ray source XR50 and PHOIBOS-100 electron analyser) measurements. STM was done with an etched tungsten tip, and XPS used the monochromated Al-K_α_ radiation source (1486.6 eV). LEED/LEEM and Secondary Electron Yield (SEY) NEXAFS were done in the LEEM/XPEEM (SMART) microscope operating at the UE49PGM undulator beamline of the BESSY II synchrotron light source at the Helmholtz Center Berlin (HZB). The aberration corrected and energy filtered LEEM-XPEEM system achieves a lateral resolution of 2.6 nm in LEEM mode.^29,30^ As a consequence of the low energy electrons involved in LEEM/LEED, both methods provide an investigation depth of only a few atomic layers, resulting in a very high surface sensitivity.^31^ On the other hand, the detection depth of SEY is yet under debate due to high deviations of the inelastic mean free path (IMFP) from the universal curve, with literature values ranging from 0.5 nm up to 3 nm for electrons with energies above the Fermi level.^32–35^ Nevertheless, it is generally accepted that NEXAFS is a more a bulk-sensitive technique in comparison with XPS. Both systems were equipped with commercial microwave plasma cracker sources (MPS-ECR-HO, SPECS GmbH), which were used to direct streams of oxygen ions at low pressures (indicated below) towards the sample. The plasma sources were mounted in UHV chambers separated by gate valves from the analysis chambers to avoid background oxygen during the subsequent surface analysis measurements.

Copper single crystals with (111) and (100) orientations (from MTI Corporation, and MaTeck) were prepared by successive cycles of sputtering with Ar^+^ ions (10^−5^ mbar) and annealing at 880 K until clean and flat surfaces were obtained. Before plasma exposure, the samples were cooled down to RT to avoid thermal oxidation. The plasma treatments and subsequent measurements were done in a sequential fashion. A sequence consisted of two steps. First, a sample was exposed to the plasma for a set amount of time. Subsequently, it was transferred to the analysis chamber (within UHV) and characterized. This order was then repeated in the next sequences without any intermediate cleaning or annealing processes. Consequently, the plasma exposure is cumulative and the sum of all treatments carried out before. The plasma sources were operated at an oxygen pressure of ∼3 × 10^−5^ mbar in the STM/XPS system and at a slightly higher pressure of ∼4 × 10^−4^ mbar in the LEEM/XPEEM system, resulting in an about 20 times faster plasma oxidation in the LEEM/XPEEM system compared to the STM system. The latter was quantitively determined by comparing the analysed oxidation rates (see Results and ESI†). The sample was placed at about 100 mm (LEEM/XPEEM) and 150 mm (STM/XPS) in front of the plasma source. We used anode voltages of 400 V, roughly translating to the kinetic energy of the extracted ions. The experimental parameters and the sample-source distance were kept constant in each system during different stages of the plasma exposure. An ion current of ∼1 μA was measured in the LEEM/XPEEM system.

While LEED, XPS and NEXAFS were done after each sequence for the entire plasma treatment, image acquisition with STM was done until the measurements became increasingly difficult due to reduced surface conductivity and growing surface roughening, resulting in increasingly drastic tip changes. We used the Gwyddion and WSxM software packages for image analysis of the STM data, and CasaXPS for the analysis of the XPS spectra.^36,37^

## Results and discussion

### Morphology

**Fig. 1:**
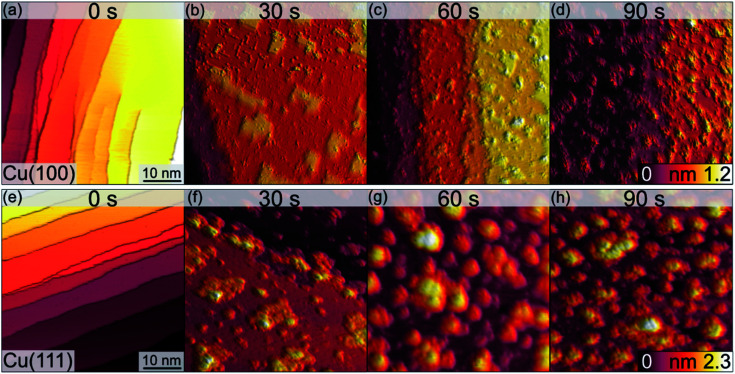
STM images of the clean (0 s) Cu(100) (a–d) and Cu(111) (e–h) surfaces and those after the initial oxide growth following the exposure to an O_2_-plasma at RT, 3 × 10^−5^ mbar, for the times indicated. The lateral sizes of all images are 50 nm × 50 nm. The height scales are cut off at 1.2 nm (top row) and 2.3 nm (bottom row) to equalize each tile. Imaging parameters for (a–d): *U* = −0.6 V to −1.5 V, *I*_t_ = 115–222 pA. Imaging parameters for (e–h): *U* = −1.5 V, *I*_t_ = 155–289 pA.

The morphological changes after sequential plasma treatments have been investigated using LEEM and STM in the two different setups. While STM allows resolving the surface in greater detail and with relevant sensitivity in height, LEEM favours the visualization of larger areas. Fig. 1 shows a series of STM images, starting from the clean Cu(100) and Cu(111) surfaces, after successive plasma exposure of 30 s at each sequence. The clean substrates show a stepped structure with no step bunches within the observation range of the instrument. Clearly, island growth sets in after the first treatment. The stepped structure of the surfaces is however retained as we can find steps in several images. We can therefore conclude that the plasma treatment is mild enough not to result in destructive changes on a scale beyond the surface structure. The oxide island coverage increases with each sequence, and qualitatively it is obvious that the island growth and nucleation progresses are faster on the Cu(111) surface as compared to Cu(100).

**Fig. 2:**
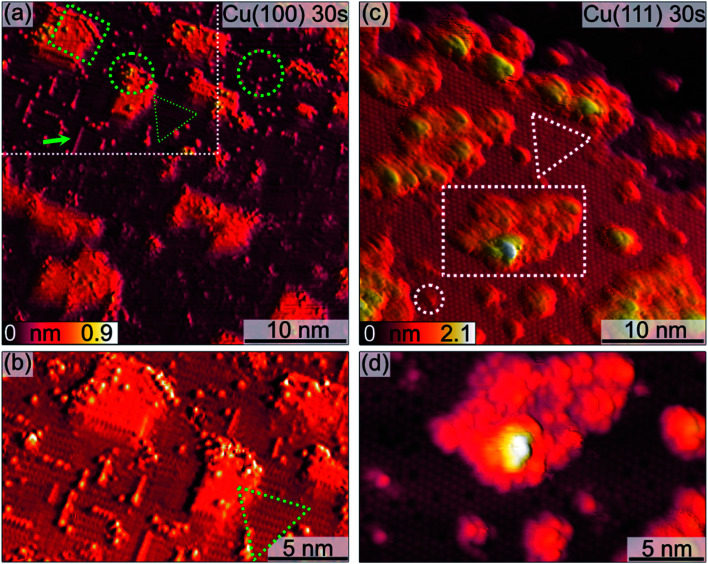
STM images of the (a, b) Cu(100) and (c, d) Cu(111) surfaces after 30 s plasma exposure at RT, 3 × 10^−5^ mbar. (a) Overview of Cu(100). (b) Area marked with the white rectangle in (a). Imaging parameters *U* = −0.6 V to −0.9 V, *I*_t_ = 155 pA. (c) Overview of Cu(111) and (d) zoom on the area around the island marked with the white rectangle in (c). Imaging parameters: *U* = −0.9 V, *I*_t_ = 115 pA. Green and white markers highlight key features of the surface morphology.

**Fig. 3:**
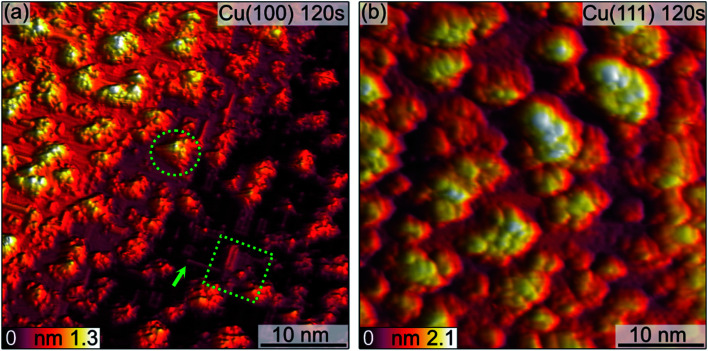
STM images of the (a) Cu(100) and (b) Cu(111) surface after 120 s plasma exposure at 3 × 10^−5^ mbar O_2_. In (a), marked with green shapes are key features discussed in the text. Imaging parameters: (a) *U* = −0.9 V, *I*_t_ = 155 pA (b) *U* = −1.5 V, *I*_t_ = 115 pA.

Fig. 2 shows a detailed view of the Cu(100) and Cu(111) surfaces after the initial 30 s plasma exposure. In Fig. 2(a), four distinct features are visible. Rectangular islands (green square) of monoatomic height and stripe-like structures (arrow) following the (100) surface orientation. The surface, including the rectangular islands, is also covered in part by small adsorbate clusters (green circles). The clean, flat substrate (green triangle) resembles a missing-row (MR) reconstruction which is a known O/Cu system following oxygen exposure.^38^ We can identify a ladder-type contrast^39^ as shown in Fig. 2(b). We find larger numbers of the small clusters nucleated at ad-islands. It has been reported that the edge and corner sites of the ad-islands are preferential nucleation sites for oxide islands.^18,19^ This is more apparent in the 120 s image in Fig. 3(a). For this greater exposure, numerous oxide clusters (green circle) are covering the surface in conjunction with the stripes (arrows) and rectangular islands (green square). However, the substrate structure itself is still seemingly intact, and the stepped surface structure is retained. In comparison, on Cu(111) we did not observe similar preferred nucleation sites. The Cu(111) surface after 30 s of plasma exposure is shown in Fig. 2(c and d). From the larger scale STM image in Fig. 2(c) it is clear that the coexistence of flat, reconstructed substrate and islands is shifted towards islands on the (111) orientation. The islands can be classified into three types, the first being larger, disordered structures (white rectangle in Fig. 2(c)), which are accompanied by two types of smaller islands (white circles). The islands are not limited to monolayer height, but appear to have grown immediately three-dimensionally. The substrate appears to be reconstructed as Cu_2_O(111)-like (white triangle), as shown in Fig. 2(d).^40–42^ Typical etching along the step edges is seen, which is known to occur on Cu(111) during oxidation.^43^ Curiously, ordered surface reconstructions have been described upon thermal treatments,^22^ whereas at RT and lower dosages, unordered surfaces have been reported,^14,18^ but we see them here in combination with disordered structures and etched steps from the initial oxide growth.

Analogous to Cu(100), further sequences of plasma exposure result in a Cu(111) surface that is covered by more islands, with increasing density and height, as is evident from Fig. 3(b). Still, flat areas between islands remain intact. This wetting behaviour is also observed for the (100) orientation. Because of the nature of the plasma, it is reasonable to expect a high reactivity of the impinging oxygen ions. Assuming immediate reaction upon impact, one could expect a randomly dispersed oxygen distribution. One would also expect similar evolution of Cu_2_O and CuO for both orientations. Our observations from STM and XPS contradict this, and we interpret the differences in the copper oxide formation and evolution as a result of the thermalization of the impinging oxygen ions, rather than immediate reaction. Thermalization and the accompanying diffusion processes of oxygen and copper atoms offer an explanation for the continued island growth after the first sequences, and also for the orientation-dependent copper oxide formation.

**Fig. 4:**
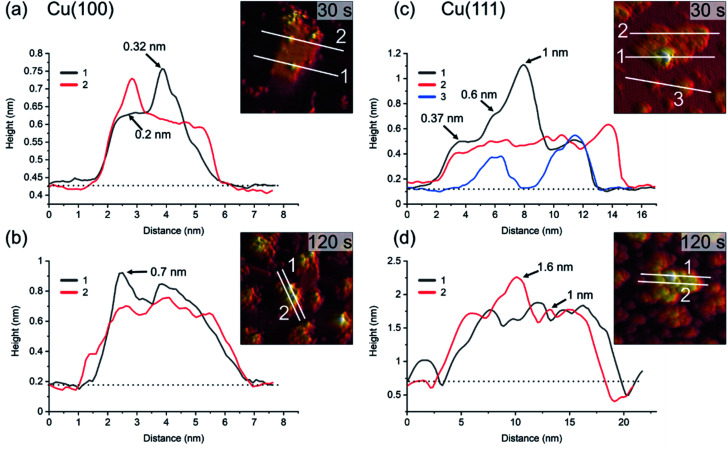
Profiles of line scans on the islands shown in Fig. 2 and 3 for 30 s and 120 s plasma exposure at 3 × 10^−5^ mbar O_2_. In (a) and (b), Cu(100). In (c) and (d), Cu(111). The insets show the position of the line scans corresponding to the graphs. The baselines (dotted) used for the determination of the apparent heights at the positions marked with arrows are also shown.

For similar O_2_-plasma exposures, the average island height on the (111) surface is higher than on the (100) surface. The observation of flat inter-insular areas is made over the entire range of STM measurements. Fig. 4 shows line scans across islands after dosing for 30 s and 120 s, respectively. One should note here that on Cu(100) we observed slight tip-induced artifacts at the bottom edges of the islands in form of a double tip, which does not resemble a real sublayer. The apparent height of the islands varies between the Cu(100) and Cu(111) substrates. The maximum and the average height of the islands is lower for islands formed on Cu(100) as compared to Cu(111), with a maximum height of ∼0.7 nm on (100) after 120 s and ∼1.6 nm on Cu(111), Fig. 4(b) and (d), respectively. In addition, the variation in height is more pronounced for Cu(111), ranging from ∼0.4 nm to 1 nm as compared to 0.2 nm to 0.3 nm on Cu(100). The height of the flat islands on Cu(100) corresponds to the step height of Cu(100). We could not identify clearly a defined height corresponding to a known step height on the islands formed on Cu(111). Specifically, for the 30 s measurement, we can see that the islands on Cu(111) have distinct step heights, possibly due to their arrangement according to the crystal structure of the oxide. We can identify two steps corresponding to the first and second island layer. One should keep in mind that these are apparent heights, since STM correlates to the local density of states which can deviate from the true height profile. Nonetheless, these results show a significant preference for a height increase of the islands in the case of Cu(111) over Cu(100).

In addition to the nanoscale morphology obtained by STM, we investigated the larger scale morphology of the samples in the LEEM/XPEEM system by analysing the low energy electrons reflected from the surface (LEEM), or, *via* X-ray photoemission electron microscopy (XPEEM), by recording the electrons emitted by the photoelectric effect. We observe in LEEM that the plasma oxidation does not change the morphology of the initial surface on scales larger than 100 nm. Both LEEM and XPEEM-NEXAFS show homogenous surfaces, with a roughness of about a few tens of nanometer that can be spatially resolved at low kinetic electron energies due to their highest sensitivity to lateral work function variations.^44^

**Fig. 5:**
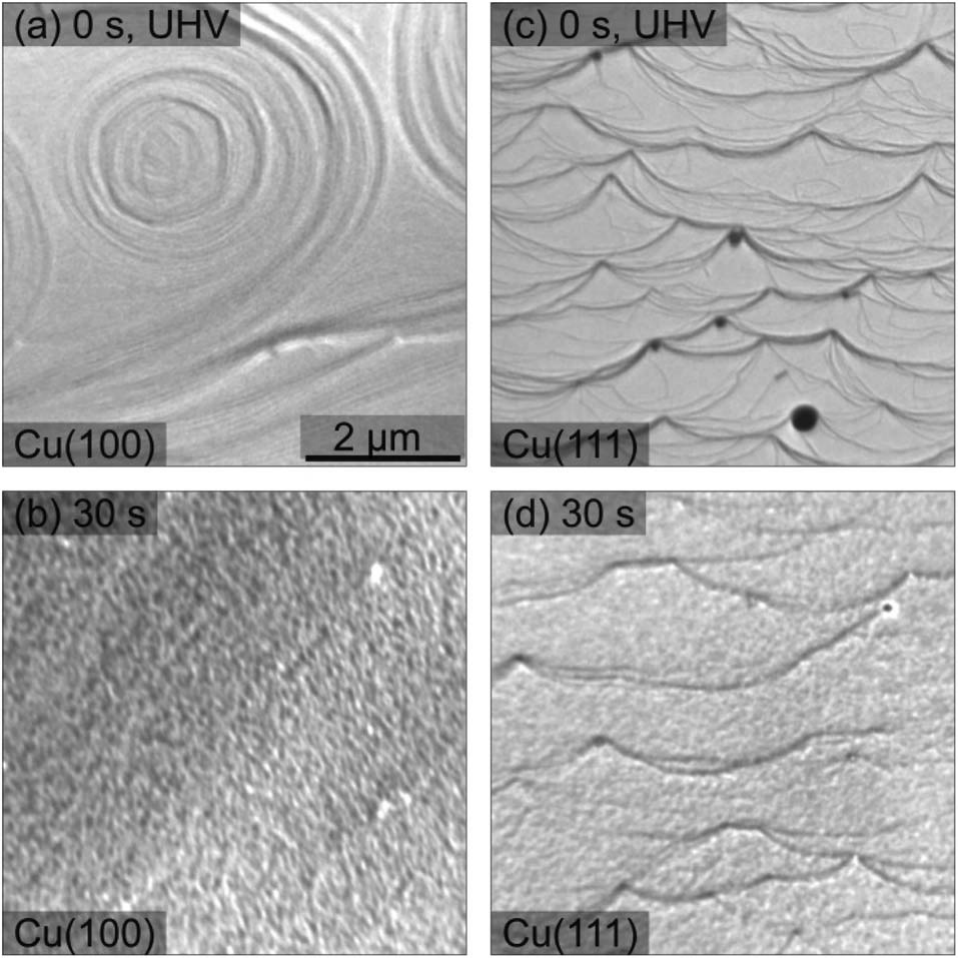
LEEM images recorded before and after 30 s of O_2_ plasma treatment of the Cu(100) and Cu(111) surfaces, top and bottom row, respectively. (a) Clean Cu(100), electron energy *E* = 20 eV; (b) Cu(100) after plasma treatment, *E* = 2.3 eV; (c) clean Cu(111), *E* = 20 eV; (d) Cu(111) after plasma treatment, *E* = 2.4 eV. The O_2_ pressure during the plasma exposure was 4 × 10^−4^ mbar. All images were taken with the same magnification shown in (a). Note that the images do not represent the same local area on the sample.

Fig. 5 presents a comparison of LEEM images acquired on clean Cu surfaces of both orientations and those exposed to an *in situ* O_2_ plasma (30 s, *p*_O_2__ = 4 × 10^−4^ mbar) treatment. On the clean surface, the atomic steps and step bunches can be identified by dark lines.^45,46^ The 30 s plasma treatment in 4 × 10^−4^ mbar O_2_ does not change the main texture significantly. However, a grainy morphology is clearly visible in Fig. 5(b) and (d), corresponding to a spatial roughening of the surface and consequently, the sharpness of the step edges and of the step bunches gets gradually lost within 30 s and in the following treatments (not shown here). This plasma-induced roughening was observed on both orientations. Additionally, by following the LEEM intensity as a function of the electron energy, one can observe work function variations upon different stages, Fig. S1 and S2.† A discussion about this aspect is presented in the ESI.†

### Crystallinity

**Fig. 6:**
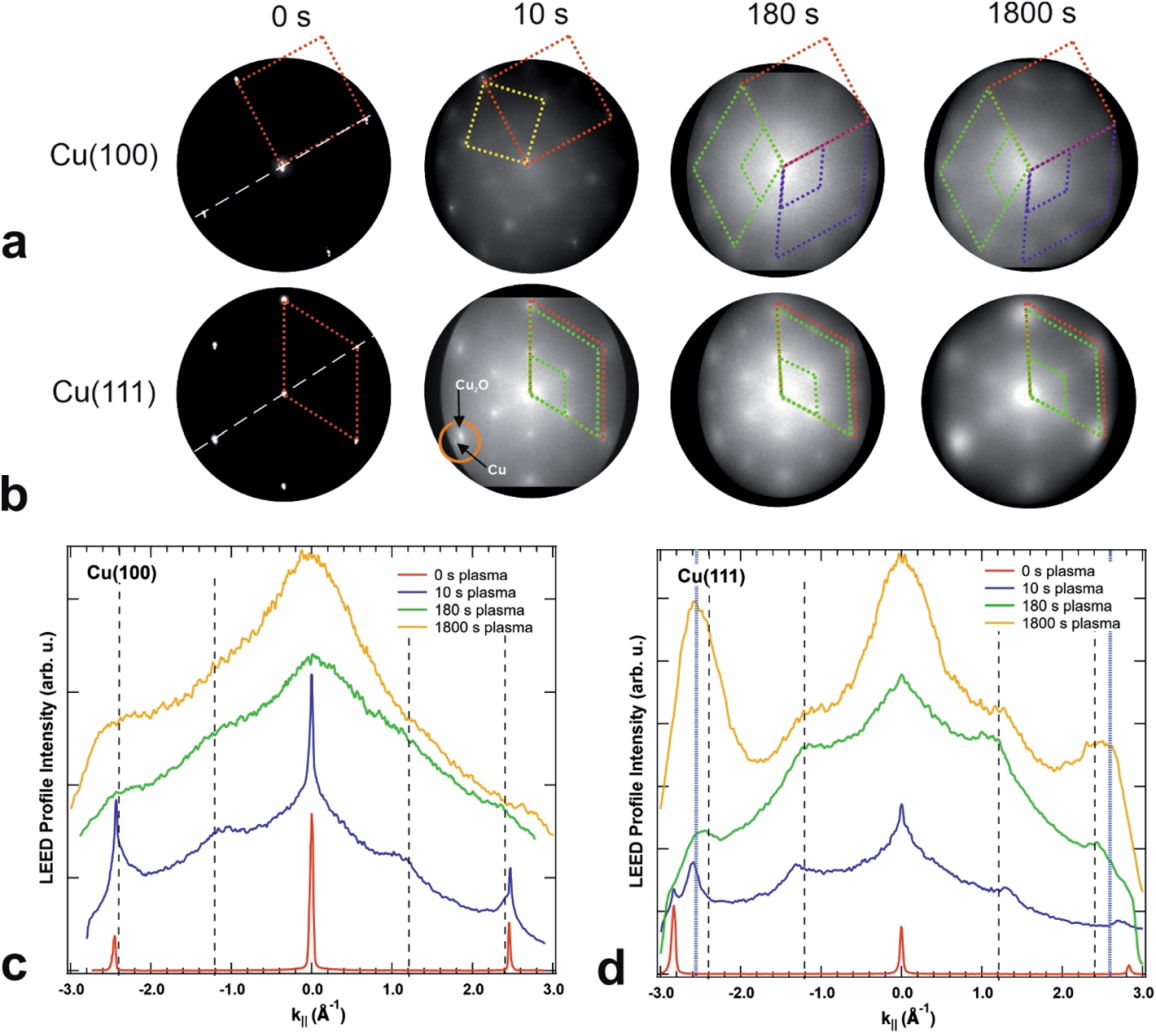
LEED images acquired on (a) Cu(100) and (b) Cu(111) after different exposures to *in situ* O_2_ plasma treatments, starting from the clean crystals (left), after 10 s, 180 s and finally after 1800 s of total oxidation time (right) performed in 4 × 10^−4^ mbar O_2_. The kinetic energy is 42 eV in all LEED patterns. The dashed lines represent the unit cells of the Cu crystals (in red), the *c*(2 × 2) reconstruction on Cu(100) (yellow square), unit cells of the two rotational domains on Cu(100) (green and purple, on top), respectively, quasi (2 × 2) reconstruction and unit cell of Cu_2_O(111) (green, at the bottom). Figures (c) and (d) present intensity profiles extracted from the LEED patterns in (a) and (b) along the directions marked by white dashed lines in the 0 s images. The vertical dashed lines mark the theoretical predicted positions of the main CuO structure and the (2 × 2) reconstruction peaks. Additional blue dotted lines in (d) mark the position of an extra spot appearing at ±2.587 Å^−1^.

The crystallinity of the surface has been characterized by LEED in the LEEM/PEEM setup after each plasma treatment. Fig. 6(a and b) presents the LEED images for the initial clean state, after 10 s, 180 s and after 1800 s of plasma treatment in 4 × 10^−4^ mbar O_2_, for both crystal orientations. The LEED patterns of the clean surfaces exhibit the spots of the metallic surface, with a four-fold symmetry for the (100) and six-fold symmetry for the (111) surface. The corresponding unit cells are presented in the pattern with red dotted lines. Within a plasma treatment of 1800 s one observes a clear change in the LEED pattern for both surfaces: (i) additional LEED spots appear – indicating a larger unit cell size in real space and (ii) the spots get increasingly blurry – exhibiting a loss in crystallinity with increasing plasma exposure. However, there are differences between the plasma oxidation of the two single crystal orientations. For both cases, the 10 s treatment constitutes a special case in the image series, since the oxide layer is incomplete and/or so thin that the LEED pattern still displays the sharp (1 × 1) spots of the partially uncovered metallic support. The LEED of the Cu(100) substrate shows additionally a *c*(2 × 2) structure (marked with a yellow unit cell) and a ring of 12 diffuse spots which becomes more pronounced upon further treatment (marked in green and purple). The *c*(2 × 2) structure is well-known for oxygen adsorbed on Cu(100), a phase that was observed during the thermal oxidation of Cu(100) at lower oxygen content, *e.g.* 0.3 ML (monolayers).^18,47^ The same 10 s treatment on the Cu(111) produced only a quasi (2 × 2) structure together with the substrate (1 × 1) spots, meaning a (111)-oriented growing oxide film, in agreement with the initial oxidation step observed in STM. A closer look exhibits a double spot structure (see the orange circle in Fig. 6(b)), which proves that the oxide layer formed has in real space a larger unit cell than the substrate. In previous studies, a mismatch of 17.5% has been estimated between the two unit cells, taking into consideration that the Cu_2_O(111) surface unit cell is 2.35 times larger than the one of Cu(111).^48^

For oxidation treatments longer than 10 s in 4 × 10^−4^ mbar O_2_, the LEED patterns do not show substrate spots anymore, but the quasi (2 × 2) superstructure with the hexagonal orientation corresponding to the ongoing growth of the Cu_2_O(111) and CuO(111) films. However, on the Cu(100) crystal, the LEED pattern is composed of 12 equally distant diffuse spots superposed to an inner smaller diffuse ring. The structure can be described by two coexisting rotational domains of hexagonal structures (see the green and purple unit cells in Fig. 6(a and b)). The same structure has been previously reported for an oxygen covered Cu(100) surface which was annealed at 870 K for a longer time,^49^ that was explained by two domains of an hexagonal phase, rotated by 90° against each other and each aligned along one crystallographic surface direction. This structure was attributed to the (111) phase of Cu_2_O, at an oxygen content of maximum 2.6 ML. In our case, the remarkable difference is that the same kind of structure could be obtained at room temperature only as a result of the interaction of the O_2_ plasma with the surface. In contrast, the LEED pattern of the Cu(111) crystal shows the formation of a single (2 × 2) domain that does not change significantly during the plasma treatment. Comparing the LEED pattern changing over time for the two crystals, one observes a spot broadening with ongoing treatment, but at the same stage of treatment the spots on the Cu(100) crystal are broader and more diffuse than on the (111). The broadening of the LEED spots in the present data set, in comparison with the previous reports,^49^ indicates a higher density of defects and smaller grain size^50^ than it would be expected for films grown by thermal oxidation. Due to the diffuse intensity in the LEED images, we present in the ESI in Fig. S3† additional LEED images recorded with 20 eV, in which the inner quasi (2 × 2) spots show higher brightness. In order to quantify the LEED data, intensity profiles along one high symmetry direction were extracted and presented in Fig. 6(c and d), together with vertical dashed lines indicating the theoretical (1 × 1) and (2 × 2) peak positions of CuO(111), *i.e.* 2.424 Å^−1^ and 1.212 Å^−1^, but it is worth to observe that Cu_2_O(111) fits in the same range, *i.e.* 2.392 Å^−1^ and 1.196 Å^−1^. One can note that in the case of the (100) crystal, the new developed wider spots appear at the theoretical values, first as shoulders in the vicinity of the Cu(100)-(1 × 1) position, *i.e.* 2.454 Å^−1^, in the case of the 10 s treatment, and later on as larger features in a background dominated curve. The gradual fading of the spots is in line also with a shift of the peaks at 1800 s towards higher values, meaning a decrease in the unit cell together with a decrease in the size of the crystalline grains. On the other hand, the oxidation of Cu(111) shows an interesting behaviour after 10 s of plasma treatment: the new diffraction spot appears at a *k*_‖_ value of 2.59 Å^−1^ (see the blue dotted lines Fig. 6(d)), that does not match the expected position of Cu_2_O and corresponds to a unit cell 7.3% smaller than the one of bulk CuO. A corresponding shift is observed also for the (2 × 2) peaks. Interestingly, the shifted (1 × 1) spot seems to be preserved after subsequent treatments, even though its contribution is contained in the overall broadening of the spot. From this analysis, one can conclude that the (111) oxide structure induced by the plasma fits better on the two directions of Cu(100), but does not preserve a good crystallinity upon longer treatments, while on the Cu(111) the structure gets compressed by forming a single kind of domain, and is therefore more stable. Based on the spot width one can estimate a grain size of about 2.5 nm for the CuO film produced by plasma-assisted oxidation, which is in good agreement with the average grain size observed by STM in Fig. 3.

From the crystallinity point of view, it is worth to compare the plasma oxidation with the thermal analogue. The latter has been well studied so far in a large range of exposure times, oxygen pressures and temperatures.^17^ In the case of the thermal oxidation of Cu(100) crystals, during the initial oxygen adsorption at low oxygen coverages of ∼0.3 ML and temperatures lower than 473 K, the *c*(2 × 2) reconstruction is observed, while at higher coverages the MR structure 
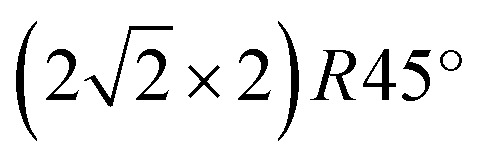
 starts to form.^51,52^ In our case, the *c*(2 × 2) structure could be identified only in the case of 10 s at 4 × 10^−4^ mbar treatment, but no MR pattern could be detected in the LEED data. Further oxygen dosing or higher temperature induce the initial growth of Cu_2_O islands that develop and coalesce. In fact, the wetting layer displays again a MR structure.^19^ Nevertheless, with the exception of the already discussed report^49^ of the two rotational domains that we observe after oxygen plasma oxidation, we could not find any other study that reports the growth of hexagonal Cu_2_O(111) on top of cubic Cu(100). In the case of the Cu(111) surface, it is known that it does not favour the adsorption of oxygen at lower coverages. Various structures and reconstructions have been reported for oxygen adsorption on Cu(111) at RT or at higher temperatures and for the initial oxidation,^24,43,53^ displaying rather complex LEED patterns. Regarding the Cu_2_O(111) reconstructions typically observed, one could identify the (1 × 1) and 
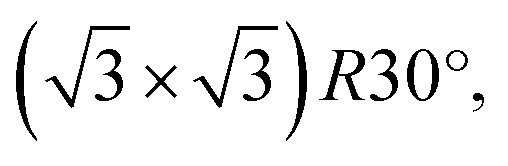
 which were attributed to a pristine oxygen-terminated (111) surface, respectively to a defective Cu_2_O(111) surface missing oxygen anions.^41,42,54^ Additionally, during the thermal oxidation, the “44” or “29” reconstructions are typically observed upon annealing at ∼423 K or ∼673 K and describe surface oxides structures with unit cells that are 44 or 29 times larger than the one of Cu(111).^25,43,55^ Other rather complex reconstructions have also been observed after exposing the Cu(111) surface to a hyperthermal oxygen molecular beam at RT.^56^ Interestingly, we could not identify an experimental study reporting a (2 × 2) reconstruction of oxygen adsorbed on Cu(111) or of Cu_2_O(111), even though there are theoretical studies that considered these kind of structures.^22^ Other studies reported a mixture of (2 × 2) and weak 
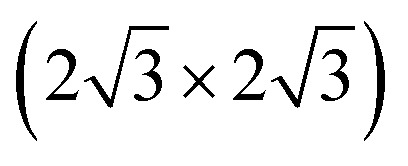
 superstructure in the case of Cu oxidation on top of Pt(111) at high temperatures,^57^ which resulted in a similar LEED pattern as the ones shown herein. Taking into consideration the spectroscopic results that will be discussed in the following section, one can assume that longer exposures to oxygen plasma produce a thick CuO layer on the surface, while the Cu_2_O is only an intermediate layer of about 1 to 2 nm thickness. One could infer therefore that the LEED data acquired for the samples exposed for longer times to the plasma correspond to a CuO(111) surface. In fact, the increase of the diffuse shape of the LEED spots with time could be interpreted as being the consequence of an increase of the lattice tension induced by the growth of the top layer. Interestingly, there are no particular reconstructions that could be observed at intermediary oxidation steps that could have indicated the formation of a different oxide. Furthermore, the attempt to anneal the crystals after the final oxidation treatment up to a maximum of ∼570 K (not shown) did not result in a stabilization of a flat oxide film, but dewetting and the formation of multiple oxide islands, where the LEED patterns of the surface did not resemble the one acquired after 1800 s of plasma oxidation in 4 × 10^−4^ mbar O_2_.

### Chemical composition

**Fig. 7:**
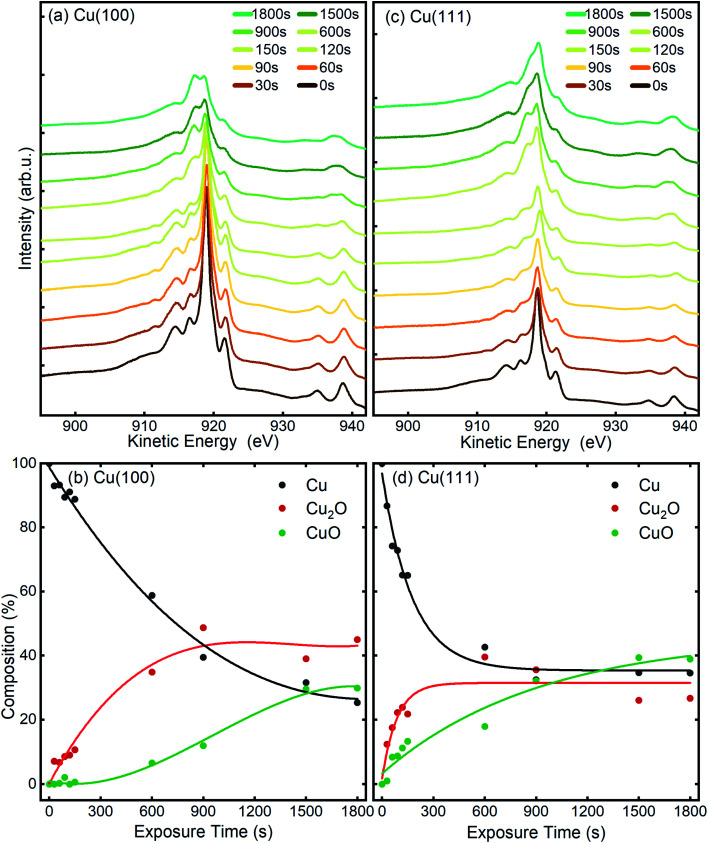
Cu LMM AES spectra measured before and after different *in situ* O_2_-plasma exposures at 3 × 10^−5^ mbar of (a), (b) Cu(100) and (c), (d) Cu(111) single crystal surfaces. The content of the different Cu species was determined by fitting and deconvolution of the Cu LMM signal (b and d). The fitted components are shown in Fig. S4 and Table S1.† The connecting lines are meant as guides for the eye.

The chemical state of the samples was probed after each step of the plasma treatment by measuring the Cu LMM Auger peak with XPS in the STM/XPS system and additionally, over longer exposure times by Cu–L edge and O–K edge NEXAFS in the LEEM/XPEEM system. The Cu LMM spectra and the related analysis of the component fitting (Fig. S4†) are shown in Fig. 7. The analysis of the Cu LMM peaks after each plasma treatment step reveals a different evolution of the content of the Cu_2_O and CuO species for the Cu(100) and Cu(111) surfaces, as shown in Fig. 7(b) and (d), respectively. Cu_2_O and CuO are formed immediately upon plasma exposure on Cu(111), in contrast to the Cu(100) surface, where only Cu_2_O is formed up to at least 150 s at 3 × 10^−5^ mbar O_2_. The ratio of metallic to oxidized copper species decreases also faster on Cu(111) for exposures under 900 s, after which both surfaces exhibit slower oxide growth. The former behaviour is in accordance with the STM morphology results, where the comparative island growth over time inferred a swifter oxidation of Cu(111). After a total exposure of 1800 s, a significant difference in the Cu species is apparent. On Cu(111), the nominal Cu_2_O content has decreased to 27% and CuO increased to 39%. On Cu(100), the fractions are reversed, with 45% Cu_2_O and 29% CuO. Indeed, we initially see that the Cu_2_O and CuO content on Cu(111) increases similarly, before the Cu_2_O contribution levels off after 150 s, while CuO still increases. This trend was different on Cu(100), where the Cu_2_O increases faster than CuO until an inflection point is reached after 900 s. The remaining detectable metallic copper after 1800 s *in situ* O_2_-plasma exposure for both crystals is similar. With 25% for Cu(100) and 34% on Cu(111), which is a sign of a thin film in the range of a few nanometers, which is compatible with the STM results. It should be noted that the XPS signal of the deeper layers (Cu, Cu_2_O) is also expected to be dampened with increasing thickness of the CuO overlayer.

**Fig. 8:**
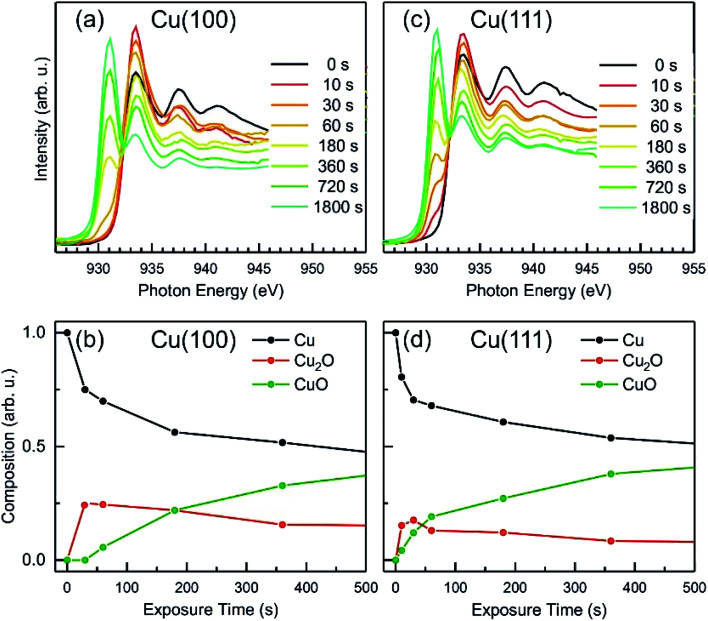
NEXAFS after *in situ* oxygen plasma treatment of Cu(100) and Cu(111) at 4 × 10^−4^ mbar. (a and c) Cu L-edge NEXAFS data at different doses. (b and d) The analysis displays the content of metallic Cu, Cu_2_O and CuO *versus* oxygen plasma treatment time.

To extract additional information on the formation and stability of the different oxide species generated upon O_2_-plasma exposure, NEXAFS spectra have been measured in microscopy mode, by recording the signal around the maximum of the secondary electrons peak, using an energy filter. No local contrast could be resolved, which proves the homogenous oxidation of the surface, and therefore, the NEXAFS spectra that are discussed herein represent the total intensity of the emitted electrons recorded in a field of view of 20 μm. In Fig. 8, the Cu L-edge NEXAFS spectra measured after each O_2_ plasma treatment are shown, as well as the intensity of various components, Cu, Cu_2_O and CuO, as determined by a linear combination (LC) analysis based on NEXAFS fingerprints of the different Cu species.^58,59^ The details regarding the LC analysis can be found in the ESI (Fig. S5(a) and S6† for Cu L-edge and with Fig. S5(b)† showing O K-edge). The variation of the intensity profiles is in a good agreement with the XPS/AES measurements described previously, considering a different signal damping for the two instrumental set-ups. In the case of Cu(100), the initial oxidation steps also show only an increase of the Cu_2_O component, while the first signal of CuO could be detected only after a total exposure of 60 s O_2_-plasma in 4 × 10^−4^ mbar. On the other hand, the spectra of the Cu(111) surface show the formation of both, Cu_2_O and CuO species right after the 10 s treatment. In both cases, after about 60 s atomic oxygen exposure, the Cu_2_O signal gradually decreases, while CuO continues to increase, which can be explained with the signal damping of the Cu_2_O underlayer caused by the CuO film overgrowth discussed in the following.

**Fig. 9:**
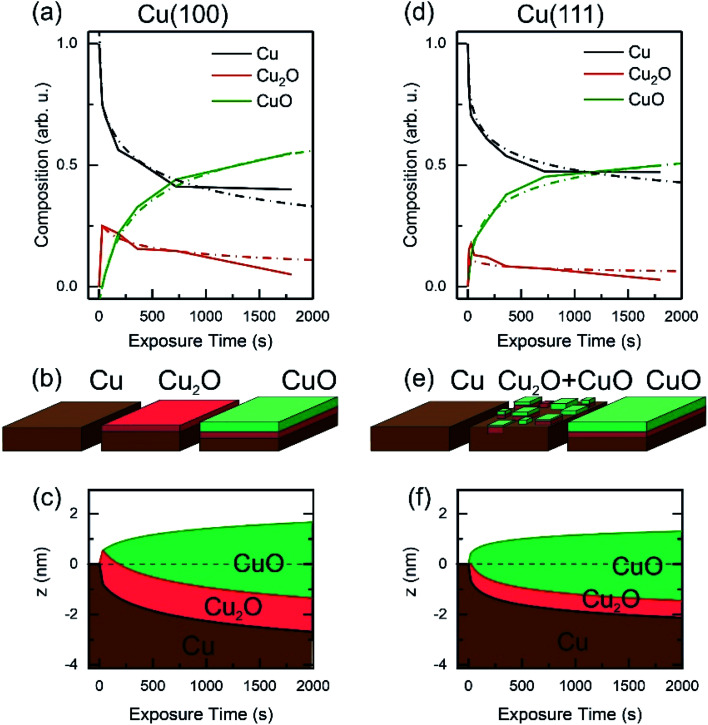
Oxide film growth on Cu(100) (a–c) and on Cu(111) (d–f) by oxygen plasma treatment at 4 × 10^−4^ mbar. (a) and (d) Raw data and fitting of the NEXAFS composition using a damping model. (b and e) Schematic of the oxidation model showing the initial metallic copper surface, the intermediate state with a complete Cu_2_O film on the Cu(100) crystal and a mixture of Cu_2_O and CuO for the Cu(111) surface. As a final state the CuO film overgrows the Cu_2_O film. Panels (c and f) exhibit the sample depth profile over the plasma exposure time using a damping model. After 30 s the Cu_2_O film keeps a constant thickness and is overgrown by the CuO film, whereas the growth rate is damped over dosage.

Based on the evolution of the intensity extracted from the NEXAFS spectra, we constructed a model to explain the oxide growth during the plasma exposure of the two crystal orientations. A simple model can be imagined in the case of the Cu(100) orientation, based on the two stages of gradual oxidation, *i.e.*, Cu → Cu_2_O → CuO, where we assume for the first stage a linear increase of the concentration of Cu_2_O species, followed by the CuO growth on top. The details about this model are described in the ESI.† We started with the assumption of a sandwich-like film structure, where the thickness of Cu_2_O species increase linearly within 30 s and stays constant at *δ* = 1.3 nm in the following. We considered the attenuation of the intensity with the thickness of the oxide layer, and tried to correlate the intensity evolution with the oxide layer thickness and with the exposure time to the oxygen plasma. Fig. 9(a) presents a fit of the Cu composition displayed in Fig. 8(b) as a function of the total time, assuming that the plasma oxidation rate is exponentially damped by the thickness of the growing oxide film, yielding 
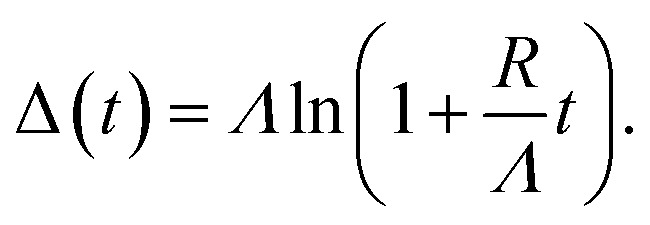
 Here, *Λ* is the effective oxidation length and *R* is the initial oxidation rate (*i.e.* thickness per time, see also ESI†). The fitting curves prove that the damping model employed largely describes the experimental curves. We also tried a model considering a linear growth of the oxide which however did not match the experimental data, which is shown in the ESI for reference, Fig. S7.† The parameters extracted from the fits are displayed in Table S2.†

By considering the inelastic mean free path to be around 3 nm at the implied electron kinetic energy, the damping model provides an initial oxidation rate of the CuO layer of *R* = 0.015 nm s^−1^ and an effective oxidation length of *Λ* = 1.05 nm. Based on these values we constructed the schematic representations shown in Fig. 9(b), where the two-step growth taking place in Cu(100) is illustrated for the two copper species as a function of the exposure time and total thickness. For the growth profiles in Fig. 9(c) we considered the different atomic Cu densities in the metallic support and the two oxide structures. This results in the oxide film growth into the Cu bulk as well as out of the surface, whereas the initial surface position is defined at *z* = 0 nm (dashed lines in Fig. 9(c)).

For the oxidation of the Cu(111) crystal, we modified the model. The two oxide species grow again in a sandwiched structure with CuO on top of Cu_2_O, but, contrary to Cu(100), (i) the CuO starts to grow from the very beginning on Cu(111) and (ii) the Cu_2_O grows linearly in thickness up to 20 s and keeps a thickness of constant *δ* = 0.66 nm in the following. Despite the uncertainty of the initial growth stages, the damping model describes the experimental data quite well and is similar to the growth on Cu(100). However, the growth parameters on Cu(111) are slightly different: the Cu_2_O film thickness is half the one of Cu(111), the oxidation rate *R* = 0.03 nm s^−1^ and the effective oxidation length of *Λ* = 0.6 nm differ by a factor of about 3 and 0.6, respectively. Notably, with these two sets of oxidation parameters, the XPS data in Fig. 7 are also well described, if mainly the mean free path length of the electrons is adapted to 1.55 nm (see ESI, Fig. S8†). Concluding from the chemical analysis, it is demonstrated that the oxide formation (at the investigated exposures) is limited to a confined space near the surface. The ratio between the different oxides depends on the exposure time, with different evolution trends for the two surface orientations.

We have shown that continued oxidation progresses at a faster rate on Cu(111) than on Cu(100), which might seem counterintuitive since Cu(100) is more open than the close-packed Cu(111) surface. However, since upon first exposures we observed Cu_2_O-like reconstructions, it is then logical that the continued growth rate is dependent on these new surfaces with different reactivities. Additionally, since Cu diffusion to the surface is governing the oxidation, we speculate that the denser Cu(111) layer provides a higher availability of copper atoms near the oxidation front.

In our oxidation study, we also find similarities and deviations to reported thermal oxidation dynamics on the two surface orientations. On Cu(100), Lahtonen *et al.* described a structure of disordered Cu_2_O islands on a reconstructed surface, which they achieved by dosing a total of 9.4 × 10^5^ L O_2_ at 3.7 × 10^−2^ mbar and 373 K.^18,19^ In STM the resulting structures appeared similar to our observations for plasma treatments at exposure times below 120 s, corresponding to a dosage of only 2700 L. However, the conditions differ greatly, as we found this to happen at a significantly lower O_2_ pressure (3 × 10^−5^ mbar) when using a plasma treatment. Critically, we also found further oxidation towards a closed CuO film for continued sequences, which has not been reported to occur in thermal oxidation processes without the use of elevated temperatures. Initial oxidation of Cu(111) at RT has been reported to coincide with the appearance of triangular oxide islands on terraces, which we have not observed here.^43,60^ A study of Cu(111) oxidation at RT *via* air injection has shown flat, fringed islands of monolayer height, which contrasts with the height increase of the islands observed here after plasma treatment.^61^ Following comparison with literature, clear distinctions between oxygen plasma treatments and other means of oxidation are apparent, as described above.

Our findings regarding the time dependence of the oxide composition allow us to rationalize previous studies of the catalytic impact of oxygen plasma treatments on copper electrocatalysts for the CO_2_RR. In a study of plasma-treated copper foils, ethylene selectivity was found to increase upon a short O_2_-plasma treatment of the Cu foil. However, longer and more intense treatments were found to be detrimental for the C_2_H_4_ yield.^9^ Our present results, revealing the formation of a Cu_2_O layer upon the initial plasma exposure, corroborate the earlier hypothesis that Cu(i) species had a positive influence on the C_2_ product selectivity during CO_2_RR. Furthermore, we also demonstrated here that further oxygen plasma treatments result in the formation of a CuO film on top of the Cu_2_O layer. Such overlayer containing Cu(ii) oxide species can negatively affect the CO_2_RR selectivity of Cu surfaces subjected to longer O_2_-plasma exposures, as seen in ref. 9. Importantly, a recent *in situ* XAS/EXAFS study shows that the presence of Cu(ii) oxides inhibits dissociative adsorption of CO_2_, a prerequisite to hydrocarbon formation, due to the preferential formation of copper carbonates that prevent effective charge transport.^62^ It was further demonstrated that on electrodes consisting of Cu(0) and Cu(i) oxide species, this hindrance is not observed and thus, hydrocarbon formation is not inhibited during CO_2_RR.

The results presented here allow the rational selection of precise O_2_-plasma parameters to control the nature of the Cu oxide formed and to tune it towards desirable Cu_2_O/Cu ratios, while avoiding the generation of Cu(ii) species. We have also established the onset and evolution of the accompanying CuO formation and revealed that the Cu surface orientation influences the oxide composition. For instance, CuO is immediately formed on Cu(111) even after very short O_2_ plasma exposure times, which implies that in order to avoid the presence of CuO in the pre-catalyst electrodes, (100) facets should be preferentially selected. Copper nanocubes constitute an ideal system to maximize (100) facets, while minimizing material use. Recently our group investigated the role of Cu oxide species electrochemically re-generated on Cu_2_O nanocubes through potential pulses during CO_2_RR.^63^ A pronounced selectivity shift from C2 to C1 products was observed, depending on the pulsing potential regime, with thin Cu_2_O/Cu interfaces being more selective for C_2_H_4_, while Cu_2_O/CuO interfaces and bulk-like Cu_2_O yielding CH_4_.

As it is shown in this work, low pressure plasma treatments can be used to controllably produce specific Cu Oxide species and surface morphologies under mild conditions that are advantageous for the selectivity control in structure/chemical state-sensitive reactions.

## Conclusion

Here, we systematically investigated the oxidation of low index copper surfaces at RT under the influence of an oxygen plasma at low pressures of 10^−5^ to 10^−4^ mbar O_2_, employing a multi-technique approach in two different setups that allowed comprehensive sample characterization while controlling for reproducibility. Our study of non-thermal plasma-assisted oxidation constitutes one of the first studies to provide insight on the resulting surface structure and composition by such plasma treatments. We revealed not only different growth behaviors of the two investigated Cu crystal orientations, but at the same time we show that longer plasma-assisted oxidation stabilizes on both substrate orientations an ordered CuO(111) film.

We identified different behaviors regarding the evolution of the morphology and oxide composition on Cu(100) and Cu(111) surfaces. The initial growth of an approximately 1.3–1.7 nm thick homogenous Cu_2_O film on the (100) substrate is similar to thermal oxidation. However, on the (111) substrate, both Cu_2_O and CuO species form simultaneously during the first plasma exposure. This behavior can be correlated not only to distinct morphological transformations, but also with a different growth rate that seems to be determined by the substrate orientation. A higher growth rate could be determined for the (111) crystal. This behavior is in a very good agreement with the STM measurements, where the closing of the oxide film proved to happen at an earlier moment on (111) as opposed to (100). On both surface orientations however, longer exposures up to 30 min lead to the development of a few nanometer thick CuO layer that shows a preferential orientation along the (111) direction, as was observed by LEED. In both cases, the CuO outer layer is interfaced with the metallic substrate by a Cu_2_O buffer, which is thinner in the case of the (111) substrate. In fact, the hexagonal (111) structure develops from the initial oxidation stages, proving that not only CuO, but also Cu_2_O prefers to grow in this particular (111) direction. Interestingly, even though the orientation of the growing oxide film is the same, the surface reconstructs distinctly in the way that the (100) substrate accommodates two different small rotational domains, rotated by 90° against each other, which has not yet been reported at RT, while the (111) develops a quasi (2 × 2) reconstruction.

These observations lead to two main implications. First, they demonstrate the ability of oxygen plasma treatments to grow predictable oxide structures at very mild conditions. This is a very useful characteristic and lends this approach to applications as a novel tool for precision synthesis of well defined metal/metal oxide interfaces. Second, our findings also emphasize the necessity to consider surface terminations when dealing with reactive environments. Even with the high reactivity of ionized oxygen, the lattice orientation of the substrate has still a major influence on the entire reaction sequence regarding nucleation, growth mode, grain size and compounds formed. Finally, our work represents an initial step towards the further exploration and utilization of plasmas for the controlled synthesis of oxide phases and tunable restructuring of surfaces.

## Data availability

The data and information supporting this article have been uploaded as part of the ESI.†

## Author contributions

S. K. and D. v. V. performed the STM and XPS experiments. S. K. analysed and evaluated the STM and XPS data. L. C. T., M. J. P., T. S., P. G., F. S. and L. d. S. C. performed the LEED, LEEM and NEXAFS experiments. L. C. T. and T. S. analysed the LEED, LEEM and NEXAFS data. S. K., L. C. T., T. S. and B. R. C. wrote the manuscript. B. R. C conceptualized and designed the study and T. S. and B. R. C. co-supervised the experiments.

## Conflicts of interest

There are no conflicts to declare.

## Supplementary Material

SC-012-D1SC04861A-s001

## References

[cit1] Mittiga A., Salza E., Sarto F., Tucci M., Vasanthi R. (2006). Appl. Phys. Lett..

[cit2] Olsen L. C., Addis F. W., Miller W. (1982). Sol. Cells.

[cit3] Reddy N. L., Emin S., Kumari V. D., Muthukonda Venkatakrishnan S. (2018). Ind. Eng. Chem. Res..

[cit4] Zhang Y., Deng B., Zhang T., Gao D., Xu A.-W. (2010). J. Phys. Chem. C.

[cit5] Hori Y., Wakebe H., Tsukamoto T., Koga O. (1994). Electrochim. Acta.

[cit6] Ma M., Djanashvili K., Smith W. A. (2015). Phys. Chem. Chem. Phys..

[cit7] Li C. W., Ciston J., Kanan M. W. (2014). Nature.

[cit8] Löffler M., Khanipour P., Kulyk N., Mayrhofer K. J. J., Katsounaros I. (2020). ACS Catal..

[cit9] Mistry H., Varela A. S., Bonifacio C. S., Zegkinoglou I., Sinev I., Choi Y.-W., Kisslinger K., Stach E. A., Yang J. C., Strasser P., Cuenya B. R. (2016). Nat. Commun..

[cit10] Scholten F., Sinev I., Bernal M., Roldan Cuenya B. (2019). ACS Catal..

[cit11] Arán-Ais R. M., Scholten F., Kunze S., Rizo R., Roldan Cuenya B. (2020). Nat. Energy.

[cit12] Dattila F., García-Muelas R., López N. (2020). ACS Energy Lett..

[cit13] Lin S.-C., Chang C.-C., Chiu S.-Y., Pai H.-T., Liao T.-Y., Hsu C.-S., Chiang W.-H., Tsai M.-K., Chen H. M. (2020). Nat. Commun..

[cit14] Hori Y., Takahashi I., Koga O., Hoshi N. (2002). J. Phys. Chem. B.

[cit15] Schouten K. J. P., Pérez Gallent E., Koper M. T. M. (2013). ACS Catal..

[cit16] Scholten F., Nguyen K.-L. C., Bruce J. P., Heyde M., Roldan Cuenya B. (2021). Angew. Chem., Int. Ed..

[cit17] Gattinoni C., Michaelides A. (2015). Surf. Sci. Rep..

[cit18] Lahtonen K., Hirsimaki M., Lampimaki M., Valden M. (2008). J. Chem. Phys..

[cit19] Lampimäki M., Lahtonen K., Hirsimäki M., Valden M. (2007). J. Chem. Phys..

[cit20] Baykara M. Z., Todorovic M., Monig H., Schwendemann T. C., Unverdi O., Rodrigo L., Altman E. I., Perez R., Schwarz U. D. (2013). Phys. Rev. B: Condens. Matter Mater. Phys..

[cit21] Dubois L. H. (1982). Surf. Sci..

[cit22] Soon A., Todorova M., Delley B., Stampfl C. (2006). Phys. Rev. B: Condens. Matter Mater. Phys..

[cit23] Wiame F., Maurice V., Marcus P. (2007). Surf. Sci..

[cit24] Navarro J. J., Tosoni S., Bruce J. P., Chaves L., Heyde M., Pacchioni G., Cuenya B. R. (2020). J. Phys. Chem. C.

[cit25] Jensen F., Besenbacher F., Lægsgaard E., Stensgaard I. (1991). Surf. Sci. Lett..

[cit26] Zheng C., Cao J., Zhang Y., Zhao H. (2020). Energy Fuels.

[cit27] Fujita K., Ando D., Uchikoshi M., Mimura K., Isshiki M. (2013). Appl. Surf. Sci..

[cit28] Stadnichenko A. I., Sorokin A. M., Boronin A. I. (2008). J. Struct. Chem..

[cit29] Schmidt T., Marchetto H., Lévesque P. L., Groh U., Maier F., Preikszas D., Hartel P., Spehr R., Lilienkamp G., Engel W., Fink R., Bauer E., Rose H., Umbach E., Freund H. J. (2010). Ultramicroscopy.

[cit30] Schmidt T., Sala A., Marchetto H., Umbach E., Freund H. J. (2013). Ultramicroscopy.

[cit31] Oura K., Katayama M., Zotov A. V., Lifshits V. G. and Saranin A. A., in Surface Science: An Introduction, Springer Berlin Heidelberg, Berlin, Heidelberg, 2003, pp. 47–75

[cit32] Ridzel O. Y., Astašauskas V., Werner W. S. M. (2020). J. Electron Spectrosc. Relat. Phenom..

[cit33] Chantler C. T., Bourke J. D. (2019). Ultramicroscopy.

[cit34] Zdyb R., Menteş T. O., Locatelli A., Niño M. A., Bauer E. (2013). Phys. Rev. B: Condens. Matter Mater. Phys..

[cit35] Nguyen-Truong H. T. (2017). J. Phys.: Condens. Matter.

[cit36] Horcas I., Fernández R., Gómez-Rodríguez J. M., Colchero J., Gómez-Herrero J., Baro A. M. (2007). Rev. Sci. Instrum..

[cit37] Nečas D., Klapetek P. (2012). Open Phys..

[cit38] Jensen F., Besenbacher F., Laegsgaard E., Stensgaard I. (1990). Phys. Rev. B: Condens. Matter Mater. Phys..

[cit39] Mönig H., Todorović M., Baykara M. Z., Schwendemann T. C., Rodrigo L., Altman E. I., Pérez R., Schwarz U. D. (2013). ACS Nano.

[cit40] Ly T. T., Lee T., Kim S., Lee Y.-J., Duvjir G., Jang K., Palotás K., Jeong S.-Y., Soon A., Kim J. (2019). J. Phys. Chem. C.

[cit41] Önsten A., Göthelid M., Karlsson U. O. (2009). Surf. Sci..

[cit42] Zhang R., Li L., Frazer L., Chang K. B., Poeppelmeier K. R., Chan M. K. Y., Guest J. R. (2018). Phys. Chem. Chem. Phys..

[cit43] Matsumoto T., Bennett R. A., Stone P., Yamada T., Domen K., Bowker M. (2001). Surf. Sci..

[cit44] Bauer E. (1994). Rep. Prog. Phys..

[cit45] Chung W. F., Altman M. S. (1998). Ultramicroscopy.

[cit46] Altman M. S., Chung W. F., Liu C. H. (1998). Surf. Rev. Lett..

[cit47] Fujita T., Okawa Y., Matsumoto Y., Tanaka K.-i. (1996). Phys. Rev. B: Condens. Matter Mater. Phys..

[cit48] Lian X., Xiao P., Yang S.-C., Liu R., Henkelman G. (2016). J. Chem. Phys..

[cit49] Devlin C. L. H., Sato Y., Chiang S. (2009). J. Appl. Phys..

[cit50] Henzler M. (1982). Appl. Surf. Sci..

[cit51] Wuttig M., Franchy R., Ibach H. (1989). Surf. Sci. Lett..

[cit52] Wuttig M., Franchy R., Ibach H. (1989). Surf. Sci..

[cit53] Judd R. W., Hollins P., Pritchard J. (1986). Surf. Sci..

[cit54] Schulz K. H., Cox D. F. (1990). Phys. Rev. B: Condens. Matter Mater. Phys..

[cit55] Skriver H. L., Rosengaard N. M. (1992). Phys. Rev. B: Condens. Matter Mater. Phys..

[cit56] Moritani K., Okada M., Teraoka Y., Yoshigoe A., Kasai T. (2008). J. Phys. Chem. C.

[cit57] Gloystein A., Nilius N. (2019). J. Phys. Chem. C.

[cit58] Greiner M. T., Jones T. E., Johnson B. E., Rocha T. C. R., Wang Z. J., Armbrüster M., Willinger M., Knop-Gericke A., Schlögl R. (2015). Phys. Chem. Chem. Phys..

[cit59] Schweinar K., Beeg S., Hartwig C., Rajamathi C. R., Kasian O., Piccinin S., Prieto M. J., Tanase L. C., Gottlob D. M., Schmidt T., Raabe D., Schlögl R., Gault B., Jones T. E., Greiner M. T. (2020). ACS Appl. Mater. Interfaces.

[cit60] Lawton T. J., Pushkarev V., Broitman E., Reinicker A., Sykes E. C. H., Gellman A. J. (2012). J. Phys. Chem. C.

[cit61] Pérez León C., Sürgers C., Löhneysen H. v. (2012). Phys. Rev. B: Condens. Matter Mater. Phys..

[cit62] Velasco-Vélez J.-J., Jones T., Gao D., Carbonio E., Arrigo R., Hsu C.-J., Huang Y.-C., Dong C.-L., Chen J.-M., Lee J.-F., Strasser P., Roldan Cuenya B., Schlögl R., Knop-Gericke A., Chuang C.-H. (2019). ACS Sustainable Chem. Eng..

[cit63] Jeon H. S., Timoshenko J., Rettenmaier C., Herzog A., Yoon A., Chee S. W., Oener S., Hejral U., Haase F. T., Roldan Cuenya B. (2021). J. Am. Chem. Soc..

