# Supplementary material for: Plasma-assisted oxidation of Cu(100) and Cu(111)
Source: Chem Sci. 2021 Oct 18;12(42):14241–53. doi: 10.1039/d1sc04861a (PMC8565394; doi:10.1039/d1sc04861a)
Supplement: SC-012-D1SC04861A-s001 [file SC-012-D1SC04861A-s001.pdf]

Electronic Supplementary Material (ESI) for Chemical Science.
This journal is © The Royal Society of Chemistry 2021

<u>Supporting Information</u>:

Plasma-assisted oxidation of Cu(100) and Cu(111)

Sebastian Kunze[a,b], Liviu C. Tănase[a], Mauricio J. Prieto[a], Philipp Grosse[a,b], Fabian Scholten[a,b], Lucas de Souza Caldas[a], Dennis van Vörden[b], Thomas Schmidt[a,*], Beatriz Roldan Cuenya[a*]

[b] *Department of Interface Science, Fritz-Haber Institute of the Max Planck Society, 14195 Berlin, Germany*

[b] *Department of Physics, Ruhr-University Bochum, 44780 Bochum, Germany*

**E-Mail: roldan@fhi-berlin.mpg.de, schmidtt@fhi-berlin.mpg.de*

Work function measurements

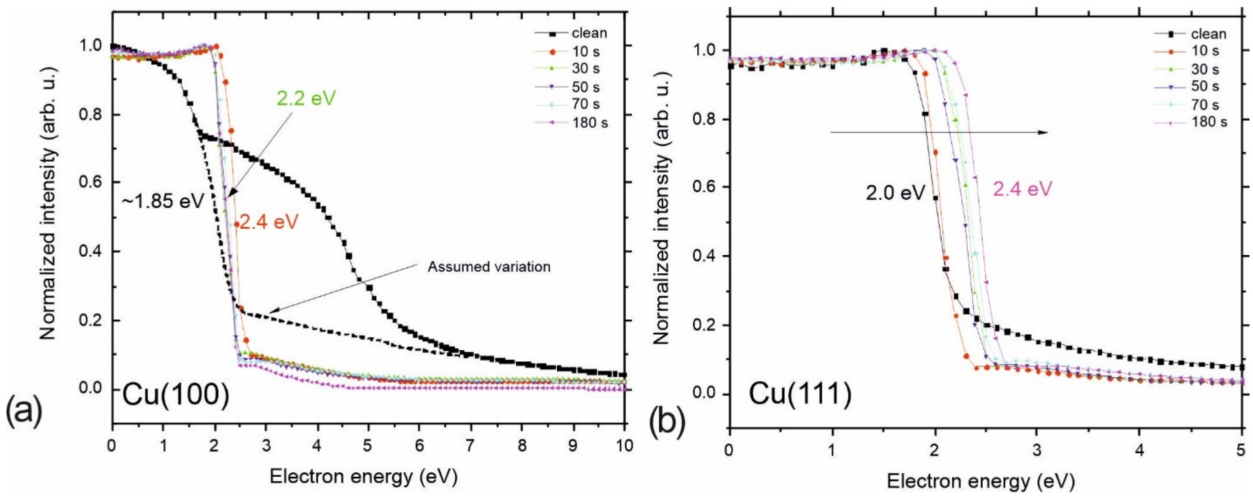

Figure S1: LEEM I-V curves showing the MEM-LEEM transition during the initial oxidation steps of a (a) Cu(100) and (b) Cu(111) single crystals. The plasma treatment was done at RT in 4×10^{-4} mbar O_2.

The LEEM I-V curves in **Fig. S1** allow the determination of the MEM-LEEM transition value, which represents the value at which the electron kinetic energy can overcome the potential energy of the surface and penetrate into the bulk. Due to this effect, the MEM-LEEM transition values are directly related with the surface. For calibration purposes, the transition energy of the clean surface is aligned to the known work function and used as reference for the oxygen-exposed surfaces.

Additionally, we determined the change in surface work function (WF) due to plasma-induced oxidation. Therefore, the image intensity has been recorded in dependence on the sample voltage (LEEM I-V) around the MEM-LEEM transition, e.g. from the voltage range where all electrons are reflected in front of the surface up to voltages where the electrons can overcome the surface potential and are partially reflected within the crystal. Considering that the MEM-LEEM transition can be correlated with the surface WF, one can track WF changes as function of the overall plasma treatment time. **Fig. S2** presents the WF variation as determined from the I-V curves represented in **Fig. S1** for both crystal orientations during the initial oxidation steps, using the WF of pristine Cu(100) at 4.65 eV[1], respectively 4.94 eV for Cu(111)[2] as references. It is important to note that the MEM-LEEM is not steep in the case of the clean Cu(100) crystal, due to the presence of a Bragg gap in the vicinity of the Fermi Level[3, 4], which might be misleading for the correct determination of the MEM-LEEM transition. In the present case, we included in **Fig. S1(a)** an additional reference curve (black dotted) of the assumed intensity variation in the absence of the signal resulting from the electron reflection on the unoccupied states, situation in which one can infer a MEM-LEEM transition of 1.85 eV for the pristine Cu(100) crystal.

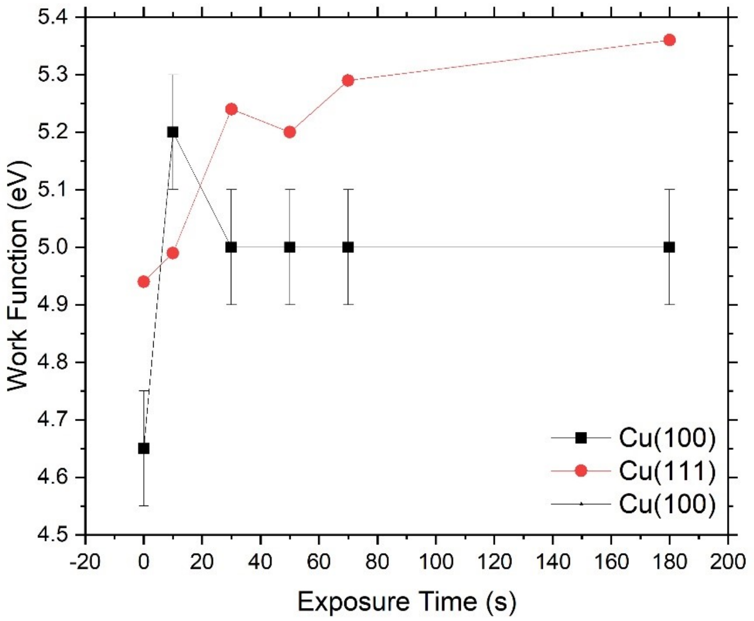

Figure S2: Work function measurements during the oxide films grown on Cu(100) (black) and Cu(111) (red)

For the two Cu surface orientations there is already a significant difference between the initial, i.e. clean state, and the first plasma treatment of 10 s in 4×10^{-4} mbar O_2. The WF suffers a shift of +0.55 eV on Cu(100), which can be correlated also with the formation of the c(2×2) superstructure, as observed in LEED (see **Fig. 6**), while the (111) crystal does not show a reasonable shift. This difference points out not only to the distinct intrinsic nature of the two orientations, but could also indicate a different initial oxidation.

Upon further dosage, the WF of Cu(100) gets stabilized to 5.0 eV. In the case of Cu(111), even though it does not show any further change after the first treatment, there is a gradual shift of +0.4 eV up to a total treatment of 180 s at 4×10^{-4} mbar O_2, which translates to a WF value of 5.36 eV, with 0.36 eV higher than the one obtained for the Cu(100) surface. The work function of the oxide surface is in good agreement with published values in the range between 5.3 and 5.5 eV[5], but values of 4.7-5.5 eV have been also reported for CuO[5]. The comparison of the two curves in **Fig. S2** proves that the gradual dosage of oxygen plasma on the two orientations with initially different WFs will induce a different behaviour regarding the WF evolution. It seems that the WF of Cu(100) is prone to stabilize after the initial oxidation, while that of Cu(111) shows a gradual change during the first stages of the plasma oxidation. This finding could be correlated with the fact that Cu(111) shows a gradual mixing of Cu_2O and CuO in the initial oxidation steps, while Cu(100) shows a slower oxidation in the first stages. The fact that the 180 s treatment is characterized by a WF difference of 0.36 eV between the two crystals is therefore not completely surprising, considering that the WF value is sensitive to the influence of various parameters such as step density, defects, adsorbates or surface reconstruction, which is in line with the other differences reported in the current study.

Supplementary LEED images

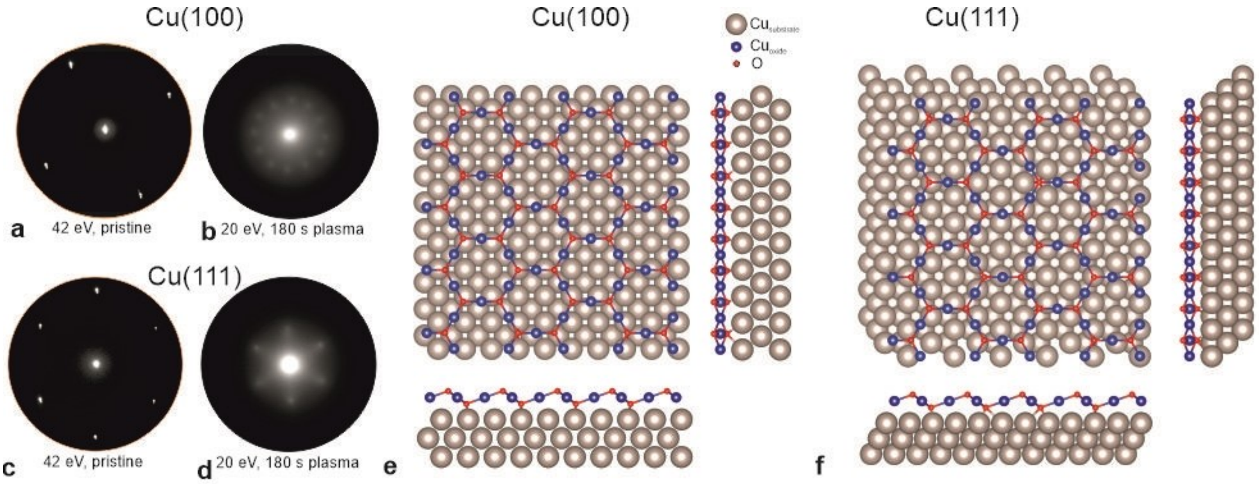

Figure S3: LEED images recorded with 42 eV on Cu(100) for the pristine sample (a), and with 20 eV after 180 s treatment (b), respectively on Cu(111) in the same conditions (c-d). Fig. (b) and (d) show a better contrast on the quasi (2×2) spots on the plasma treated samples. Nevertheless, due to the low energy involved, the size of the Ewald sphere is decreased and the LEED image does not contain the fundamental spots corresponding to the (1×1) structure. For the same reason, the LEED images measured with 20 eV on the pristine crystal (not shown) display only the (00) spot. (e-f) Structural models displaying the surface growth of $Cu_2O(111)$ on top of the two Cu model surfaces; for simplicity only one layer is shown.

X-ray Photoelectron Spectroscopy (XPS)

In order to quantify the composition of the plasma treated surfaces, we performed peak fitting of the Cu LMM spectra on a Shirley background based on the procedure and constraints outlined in *Biesinger, M. C. (2017).*[6] The obtained fit curves and Cu LMM data are shown in **Fig S4**. The binding energies were references to the most intense LMM peak corresponding to metallic Cu. We used the seven most intense peaks of metallic Cu, and four peaks for Cu_2O and CuO each. The fitting parameters for the fitting model are listed in table S1. The peak shapes are modelled with mixed Gaussian-Lorentzian (GL) line shapes with a 30:70 weight. One should note that quantification of copper in different oxidation states is quite challenging, and that the overall trend of the oxidation process of the two differently-oriented surfaces is the aspect of interest here.

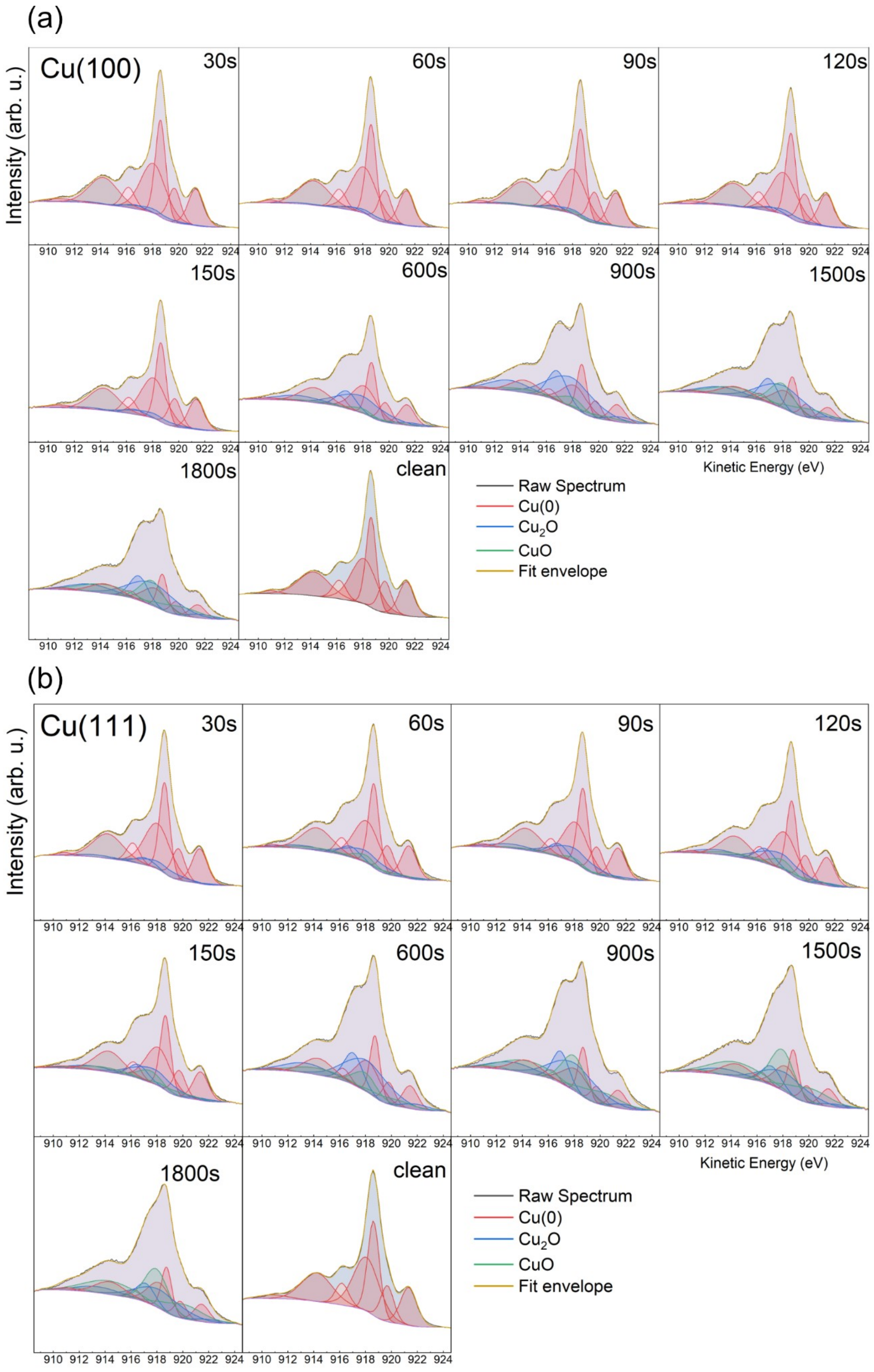

Figure S4: Cu LMM spectra and fitted components of (a) Cu(100) and (b) Cu(111) after a room temperature O_2-plasma treatment ($3{\times}10^{-5}$ mbar O_2) for the times indicated and the clean surfaces.

Table S1: Fitting parameters for the fitting model of the Cu LMM deconvolution shown in Fig. S4. The parameters of position, position constraints (Δ Position) in respect to the previous peak position, full width half maximum (FWHM) and FWHM variation (ΔFWHM) are shown.

Component	Position (eV)	Δ Position (eV)	FWHM	ΔFWHM
Cu(0)	916.24		1.2	0.2
Cu(0)	921.39	5.15	1.4	0.3
Cu(0)	919.74	1.65	1.0	0.1
Cu(0)	918.68	1.06	0.9	0.25
Cu(0)	918.13	0.43	2.3	0.2
Cu(0)	914.30	3.95	2.5	0.4
Cu(0)	910.98	3.32	1.6	0.3
Cu2O	921.64		2.3	0.4
Cu2O	917.85	3.79	4.2	0.4
Cu2O	916.76	1.09	1.6	0.3
Cu2O	913.09	3.67	3.9	0.4
CuO	920.02		3.8	0.2
CuO	917.94	2.08	2.1	0.2
CuO	914.30	3.64	4.5	0.3
CuO	911.45	2.85	2.4	0.25

Cu L-edge and O K-edge NEXAFS

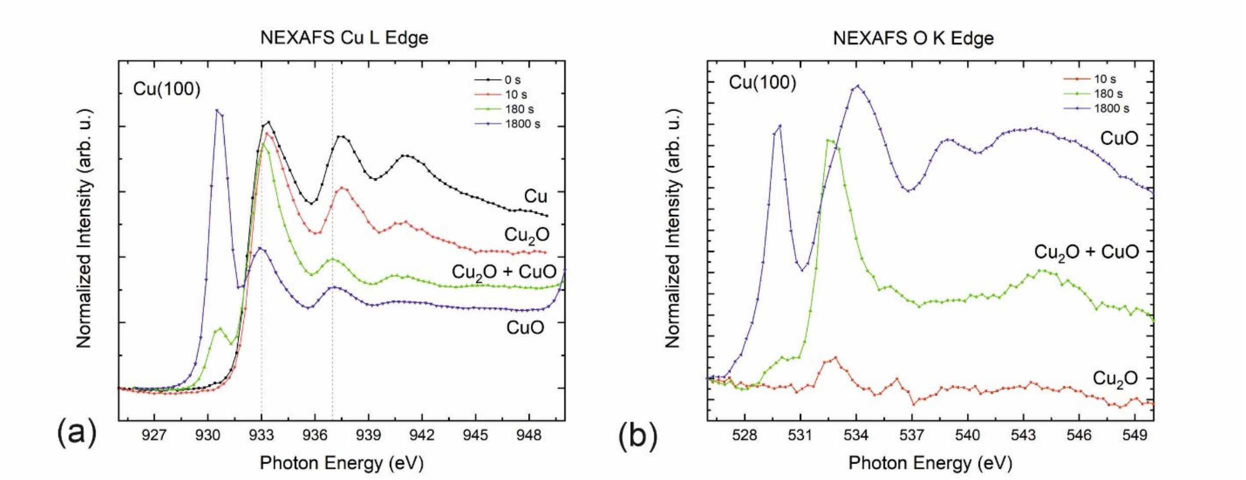

Figure S5. NEXAFS spectra measured at different stages of plasma treatment. (a) Cu L-edge, respectively (b) O K-edge NEXAFS spectra of the initial metallic surface and after 10 s, 180 s and 1800 s of plasma oxidation at 4×10^{-4} mbar of O_2, proving characteristic fingerprints of different copper oxide species. In the case of image (b), the spectrum measured for the clean sample was used for the normalization of the files, and is therefore not shown.

Composition analysis of NEXAFS spectra

The analysis of the Cu L-edge NEXAFS spectra implies that the initial state of the crystal is metallic and that the further spectra are a linear combination of characteristic spectra of metallic copper (Cu^0), Cu_2O (Cu^+) and CuO (Cu^{2+}) at a certain exposure time *t*:

$$I(t) = \sum_{i=1}^{3} \alpha_i(t) I_i(Cu_i) = \alpha_1 I_1(Cu^0) + \alpha_2 I_2(Cu^+) + \alpha_3 I_3(Cu^{2+}) \qquad (1),$$

where α_i represents a normalized weighting factor at a certain time, such that $\alpha_1 + \alpha_2 + \alpha_3 = 1$. They correspond to the “composition” of Cu^0, Cu^+ and Cu^{2+} in the spectra shown in **Fig. 8 (a)** and **(c).** The individual component spectra have been extracted from the actual data, as following: (i) the Cu^0 curve corresponds to the NEXAFS spectrum recorded on the pristine crystal; (ii) based on equation (1) the Cu^+ curve is produced as a difference between the NEXAFS spectrum measured at 30 s and the weighted pristine one, whereas the weighting factor $\alpha_2 = 1 - \alpha_1$ is choosen in a way that the residual oscillations at 938 eV and 941.5 eV (characteristic for metallic copper) are cancelled out; (iii) correspondingly, the Cu^{2+} curve is produced as a difference between the NEXAFS spectrum measured at 1800 s and the sum of weighted Cu^0 and Cu^+ curves with weighting factors α_1, α_2 and $\alpha_3 = 1 - \alpha_1 - \alpha_2$ in a way that the characteristic peak at 931 eV is well described.

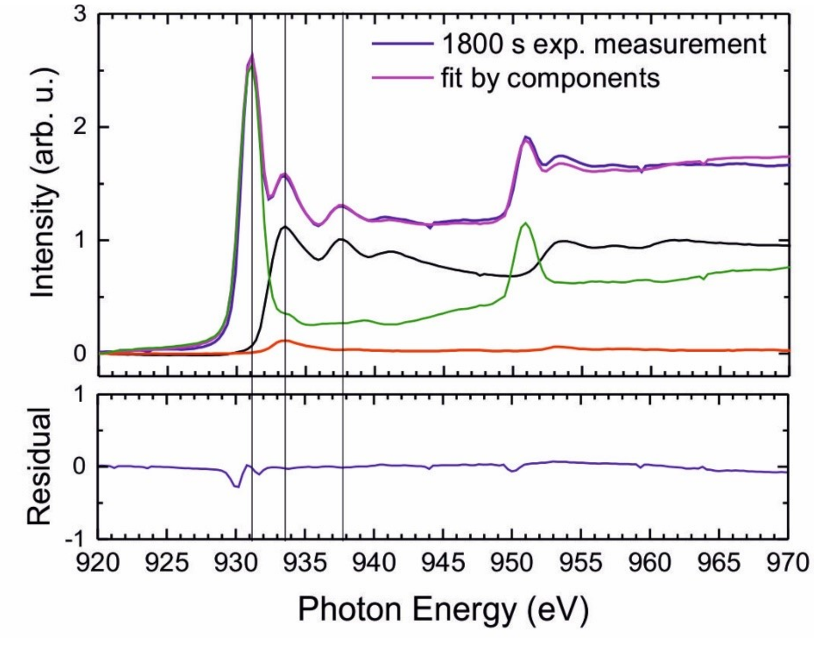

Figure S6: Example of the fit components of a Cu L-edge NEXAFS. The experimental curve (blue) is fitted by a sum of the NEXAFS components of Cu^0, Cu^+ and Cu^{2+} (black, red and green curves, respectively). The residual in the bottom exhibits the quality of the fit, whereas the emphasis is put on the range of the first three maxima. The data shows the NEXAFS measurement of the Cu(111) surface after an overall 30 min treatment with oxygen plasma.

Model of Copper Oxide Formation for Cu(100)

The model presented in the main text assumes that Cu_2O grows after the initial oxidation steps until a certain thickness δ is reached, above which the growth is continued for a Δ thickness as CuO.

Starting with the assumption that each Cu atom from the depth z contributes with an intensity $I_0 e^{-\frac{z}{\Gamma}}$, it yields the following terms for the three contribution:

$$I_{Cu^{2+}} = n_{2+} I_0\left(1 - e^{-\frac{\Delta}{\Gamma}}\right) \quad (2)$$

$$I_{Cu^{+}} = n_{+} I_0\left(1 - e^{-\frac{\delta}{\Gamma}}\right)e^{-\frac{\Delta}{\Gamma}} \quad (3)$$

$$I_{Cu^{0}} = n_0\, I_0\, e^{-\frac{(\delta + \Delta)}{\Gamma}} \quad (4),$$

where Γ represents the mean free path length of the electrons at the detected energy which is assumed to be the same for all three copper species. n_0, n_+ and n_{2+} are atomic concentrations of copper in the metallic, the Cu_2O and CuO phase. The intensity I_0 is assumed to be identical for all Cu atoms.

The normalization of the intensity eq. (2)-(4) with the total intensity

$$I_{total} = I_{Cu^{0}} + I_{Cu^{+}} + I_{Cu^{2+}} \quad (5)$$

yields in the three normalized intensities which describe the intensity composition of **Figure 9(a)** and **(d)** and is used in formula eq. 1.

$$\alpha_{Cu^{2+}} = {I_{Cu^{2+}}}/{I_{total}} \quad (6)$$

$$\alpha_{Cu^{+}} = {I_{Cu^{+}}}/{I_{total}} \quad (7)$$

$$\alpha_{Cu^{0}} = {I_{Cu^{0}}}/{I_{total}} \quad (8)$$

It is to be noted that the sum of the three components gives:

$$\alpha_{Cu^{0}} + \alpha_{Cu^{+}} + \alpha_{Cu^{2+}} = 1 \quad (9)$$

Correlation between film thickness and plasma exposure time

The growth of the Cu_2O film can be described as a linear increase between 0 to thickness δ within 30 s (at 4×10^{-4} mbar O_2), the value above which the thickness remains constant. The CuO starts to grow on top from t = 30 s on. The time dependence of the Δ thickness of this growing CuO film might be described by two models:

(1) Simple linear model: $\Delta(t) = R\times t$, with R is the constant oxidation rate of CuO
(2) Damping model, in which the oxidation rate is damped by the thickness of the film

In the both cases, the damping of the composition in **Fig. 9(a)** and **(d)** is fitted over the full exposure time of 1800 s (at $p(O_2)= 4\times10^{-4}$mbar). We make the remark that it was not yet considered that the copper layers expand in volume when the oxygen is incorporated into copper, which means that the oxide grows into the bulk, but also increases the height (see next subsection).

The damping model considers that the film grows with dosage according to the following rate equation:

$$\frac{d\Delta}{dt} = R\, e^{-\frac{\Delta}{\Lambda}} , \qquad (10)$$

where R is the initial oxidation rate, *i.e.* the oxidized film thickness per time. Nevertheless, the oxidation is hindered when the film gets thicker. The damping of the process is described by the exponential factor with Λ being the effective oxidation length. Considering the starting condition $\Delta(t = 0\ s) = 0$ nm, i.e. no oxide film in the beginning, the solution of the rate equation is:

$$\Delta(t) = \Lambda\, ln\left(1 + \frac{R}{\Lambda}t\right) \qquad (11)$$

For $Rt \ll \Lambda$ or small values of t, this formula can be approximated as $\Delta(t) \cong Rt$, what is just the expected initial linear behaviour.

Figure S7 shows clearly that fitting with the damping model describes the time dependence of the NEXAFS components much better than the linear model.

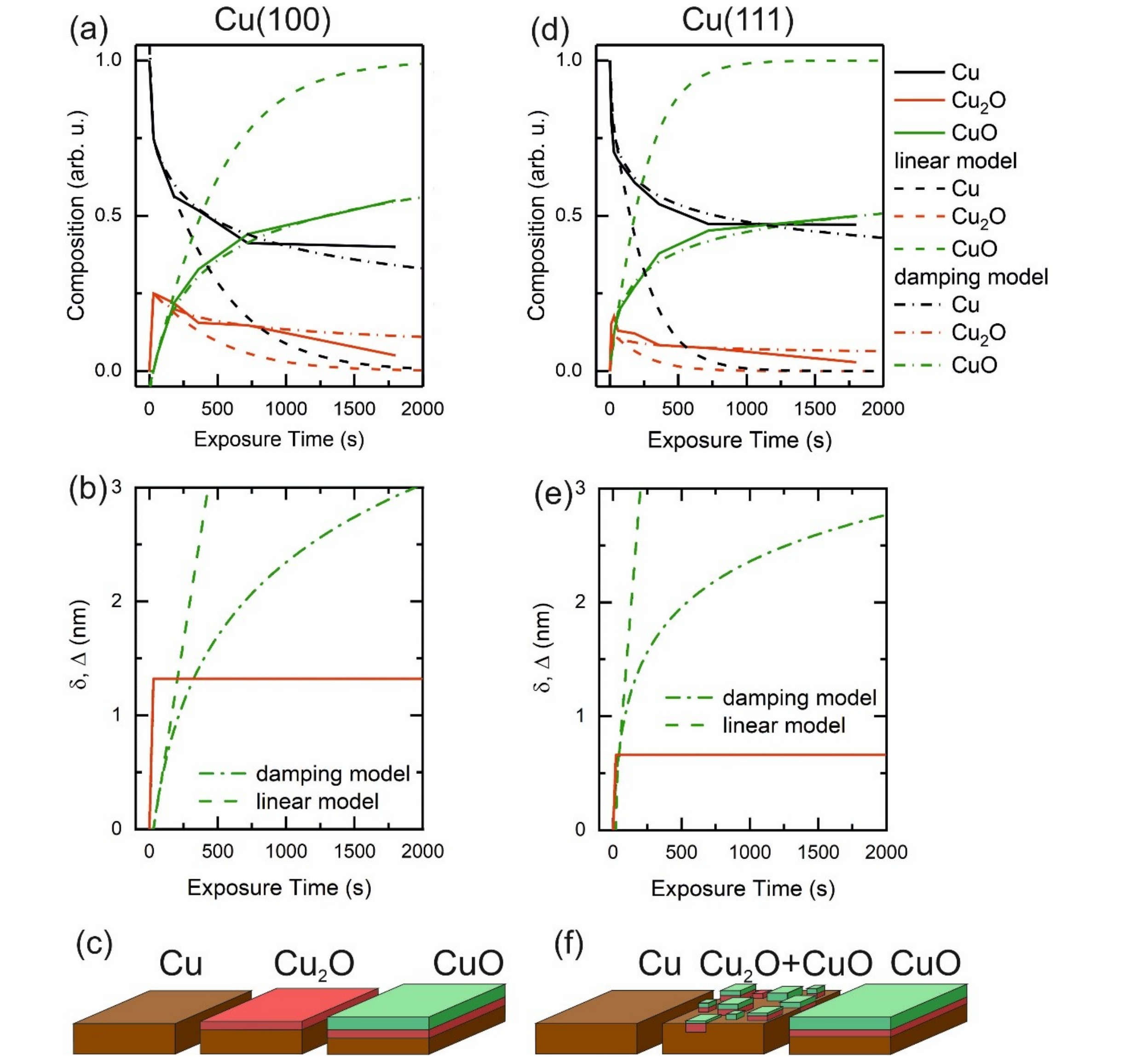

Figure S7: (a) and (d) Fitting of time dependence of the NEXAFS components with the linear (dashed lines) and the damping models (dashed dotted lines) for plasma-treated Cu(100) and Cu(111), left and right column respectively. The plasma treatment was done at 4×10^{-4}mbar O_2. (b) and (e) display the thickness of the growing CuO (red) and Cu_2O (green) film. A schematic model for the film growth is given in the bottom. The fitting parameters are listed in **Table S2.**

Table S2: Parameters extracted from the fitting of the composition evolution of Cu, Cu_2O and CuO species as function of plasma exposure time at room temperature at 4×10^{-4} mbar of oxygen for Cu(100) and Cu(111). Fits are displayed in **Fig. S7**.

	**Linear model:** $\Delta(t) = Rt$	**Damping model:** $\Delta(t) = \Lambda\, ln\left(1+\frac{R}{\Lambda}t\right)$
**Cu(100)**		
Mean free path length[7, 8] is set to	$\Gamma = 3$ nm	$\Gamma = 3$ nm

Thickness of buried Cu_2O layer	δ = 1.3 nm	δ = 1.3 nm
Initial oxidation rate of CuO	R = 0.0075 nm/s	R = 0.009 nm/s
Effective oxidation length (damping)	N.A.; $\Lambda = \infty$	$\Lambda = 1.05\ nm$
**Cu(111)**		
Mean free path length is set to	$\Gamma = 3$ nm	$\Gamma = 3$ nm
Thickness of buried Cu_2O layer	d = 0.66 nm	d = 0.66 nm
Initial oxidation rate of CuO	R = 0.015 nm/s	R = 0.03 nm/s
Effective oxidation length (damping)	N.A.; $\Lambda = \infty$	$\Lambda = 0.6\ nm$

In the literature, a wide range of IMFP values can be found depending on the material and also on the experimental or theoretical model. Therefore, for simplicity, we set one value for the IMFP, independently of the material, but from the kinetic energy. Based on this, we assume an absolute accuracy better than a factor 2 for the film thicknesses and the related growth rates. However, the relative difference between Cu(100) and Cu(111) is not affected by this because the same model is applied in both cases.

Position of the Oxide Film Boundaries

When the copper gets oxidized the atomic concentration decreases from $n_0 = 84.232\ nm^{-3}$ down to $n_+ = 50.740\ nm^{-3}$ and $n_{2+} = 52.731\ nm^{-3}$ for Cu_2O and CuO respectively (see **Table S4**). Because the amount of copper atoms is conserved, the crystal has to grow in height during oxidation (see **Figure 9 (c)** and **(f)**). If the height of the initial copper surface is defined as $= 0$, then the position of the boundary $z_{Cu^0 \| Cu^+}$ between metallic Cu and the Cu_2O, the boundary $z_{Cu^+ \| Cu^{2+}}$ between Cu_2O and CuO and the top level of the oxide film (boundary to vacuum) z_{top}

$$z_{Cu^0 \| Cu^+} = -\left(\frac{n_+}{n_0}\delta + \frac{n_{2+}}{n_0}\Delta\right) \tag{12}$$

$$z_{Cu^+ \| Cu^{2+}} = z_{Cu^0 \| Cu^+} + \delta = \left(\left(1 - \frac{n_+}{n_0}\right)\delta - \frac{n_{2+}}{n_0}\Delta\right) \tag{13}$$

$$z_{top} = z_{Cu^0 \| Cu^+} + \delta + \Delta = \left(\left(1 - \frac{n_+}{n_0}\right)\delta + \left(1 - \frac{n_{2+}}{n_0}\right)\Delta\right) \tag{14}$$

Whereas the bottom level $z_{Cu^0 \| Cu^+}$ of the oxide film is always below the level of the initial surface (i.e. $z_{Cu^0 \| Cu^+} < 0$), the top level z_{top} is always above the initial surface level (i.e. $z_{top} > 0$). The boundary between Cu_2O and CuO is first above, and at further oxidation below the initial surface level.

Composition fit of XPS data

Analogous to the fit of the NEXAFS data, the XPS composition data were fitted with the damping model. **Fig. S7** shows the experimental data with the model fit.

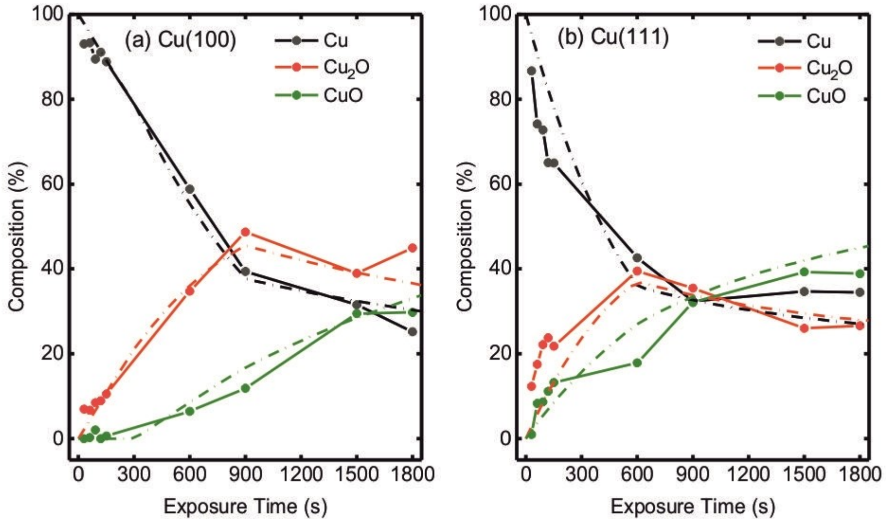

Figure S8: XPS data fit with the same model applied for the NEXAFS data. The plasma treatment was done at 3×10^{-5}mbar O_2. The fitting parameters are given in **Table S3**.

Table S3: Parameters extracted from the fitting of the composition evolution of Cu, Cu_2O and CuO species as function of exposure time at 3×10^{-5}mbar of oxygen for Cu(100) and Cu(111). Fits are displayed in **Fig. S8**.

	**Damping model:** $\Delta(t) = \Lambda \ln\left(1+\frac{R}{\Lambda}t\right)$
**Cu(100)**	
Mean free path length is set to	$\Gamma = 1.55$ nm
Thickness of buried Cu_2O layer	$\delta = 1.7$ nm
Initial oxidation rate of CuO	R = 0.0006 nm/s
Effective oxidation length (damping)	$\Lambda = 1.36\ nm$
**Cu(111)**	

Mean free path length[9] is set to	$\Gamma = 1.55$ nm
Thickness of buried Cu_2O layer	$\delta = 1.55$ nm
Initial oxidation rate of CuO	R = 0.0016 nm
Effective oxidation length (damping)	$\Lambda = 0.6\ nm$

Considering that the results presented in Table S2 and Table S3 have been determined from data measured in two different systems, one can calculate a rough calibration of the two plasma sources involved in the study. The initial oxidation rates of CuO, applying the damping model equal R = 0.009 nm/s for Cu(100), and R = 0.03 nm/s for Cu(111) for the LEEM/XPEEM and R = 0.0006 nm/s for Cu(100) and R = 0.0016 nm/s for Cu(111) for the STM/XPS. This yields a higher oxidation rate in the LEEM/XPEEM system (about 20 times higher) as compared to the STM/XPS system. This factor can be very well explained by the pressure ratio of a factor 13, together with the different sample to source distances and slight differences in the individual sources. Additionally, the different pumping speeds of the systems might play a role, resulting in a different pressure in the plasma chamber, though the nominal measured pressure in the vacuum chamber is nearly the same.

Structure

Table S4: Comparison of the crystal parameters for the metallic Cu crystal, cuprite Cu_2O and tenorite CuO.

	**Space group**	**Lattice parameter (Å)**	**Volume unit cell (Å³)**	**n_{Cu} Cu density (1/nm³)**	**a_{NN} of Cu (Å)**	**d row distance on (111) (Å)**	**Ratio to a_{NN} (Cu)**	**reference**
**Cu**	Fm-3m [225] Cubic	a=b=c=3.621 Å<br>α=β=γ= 90°<br>fcc, 4 Cu atoms per unit cell	47.488	84.232	2.561	2.218	1	https://materialsproject.org/materials/mp-30/#<br>DOI: 10.17188/1204433
**Cu_2O**	Pn-3m [224] Cubic	a=b=c=4.288 Å<br>α=β=γ= 90°<br>4 Cu atoms on fcc position<br>2 O atoms on bcc position	78.833	50.740	3.032	2.626	1.184	https://materialsproject.org/materials/mp-361/<br>DOI: 10.17188/1207131
**CuO**	Fm-3m [225] cubic	a=b=c=2.993 Å<br>α=β=γ= 60°	18.964	52.731	2.993	2.592	1.169	https://materialsproject.org/materials/mp-14549/<br>DOI: 10.17188/1190720

References

1. C. Schmidt, A. Witt and G. Witte, *J Phys Chem*, 2011, **115**, 7234-7241.
2. B. Cook, A. Russakoff and K. Varga, *Appl Phys Lett*, 2015, **106**, 211601.
3. J. I. Flege, A. Meyer, J. Falta and E. E. Krasovskii, *Physical Review B*, 2011, **84**, 115441.
4. J. I. Flege and E. E. Krasovskii, *physica status solidi (RRL) - Rapid Research Letters*, 2014, **8**, 463-477.
5. J. Deuermeier, H. J. Liu, L. Rapenne, T. Calmeiro, G. Renou, R. Martins, D. Munoz-Rojas and E. Fortunato, *Apl Materials*, 2018, **6**, 096103.
6. M. C. Biesinger, 2017, **49**, 1325-1334.
7. H. T. Nguyen-Truong, *J Phys Condens Matter*, 2017, **29**, 215501.
8. O. Y. Ridzel, V. Astašauskas and W. S. M. Werner, *Journal of Electron Spectroscopy and Related Phenomena*, 2020, **241**, 146824.
9. M. P. Seah and W. A. Dench, *Surface and Interface Analysis*, 1979, **1**, 2-11.
